# Current and Emerging Therapies for Targeting the ERK1/2 & PI3K Pathways in Cancer

**DOI:** 10.3390/ijms26178696

**Published:** 2025-09-06

**Authors:** Ethan Abizadeh, Eli Berglas, Aaron Abizadeh, Julia Glatman, Aaron B. Lavi, Mark Spivak, Tzuriel Sapir, David Shifteh

**Affiliations:** 1 College of Medicine, SUNY Downstate Health Sciences University, Brooklyn, NY 11203, USA; ethan.abizadeh@downstate.edu (E.A.); eli.berglas@downstate.edu (E.B.); julia.glatman@downstate.edu (J.G.); aaron.lavi@downstate.edu (A.B.L.); mark.spivak@downstate.edu (M.S.); 2 Zucker School of Medicine, Hofstra University, Hempstead, NY 11549, USA; aabizadeh1@pride.hofstra.edu; 3 Perelman School of Medicine, University of Pennsylvania, Philadelphia, PA 19104, USA

**Keywords:** EGFR, FGFR3, RAS, RAF, MEK, ERK, PI3K, AKT, mTOR, PTEN

## Abstract

The ERK1/2 and PI3K signaling pathways play important roles in cellular proliferation, survival, differentiation, and metabolism. In cancer, these pathways are frequently dysregulated and overactivated, resulting in poor patient prognosis and resistance to treatment. These pathways are activated by receptor tyrosine kinases and send downstream signals to effectors such as RAS, RAF, MEK, AKT, and mTOR. In this review, we highlight the key components of the ERK1/2 and PI3K pathways, the roles they play in tumor progression, and the development of inhibitors and combination therapies designed to enhance therapeutic outcomes and address treatment resistance. Our review demonstrates the need and promise for future research and clinical trials for inhibitors and combination therapies for the ERK1/2 and PI3K pathways in cancer.

## 1. Introduction

**Figure 1:**
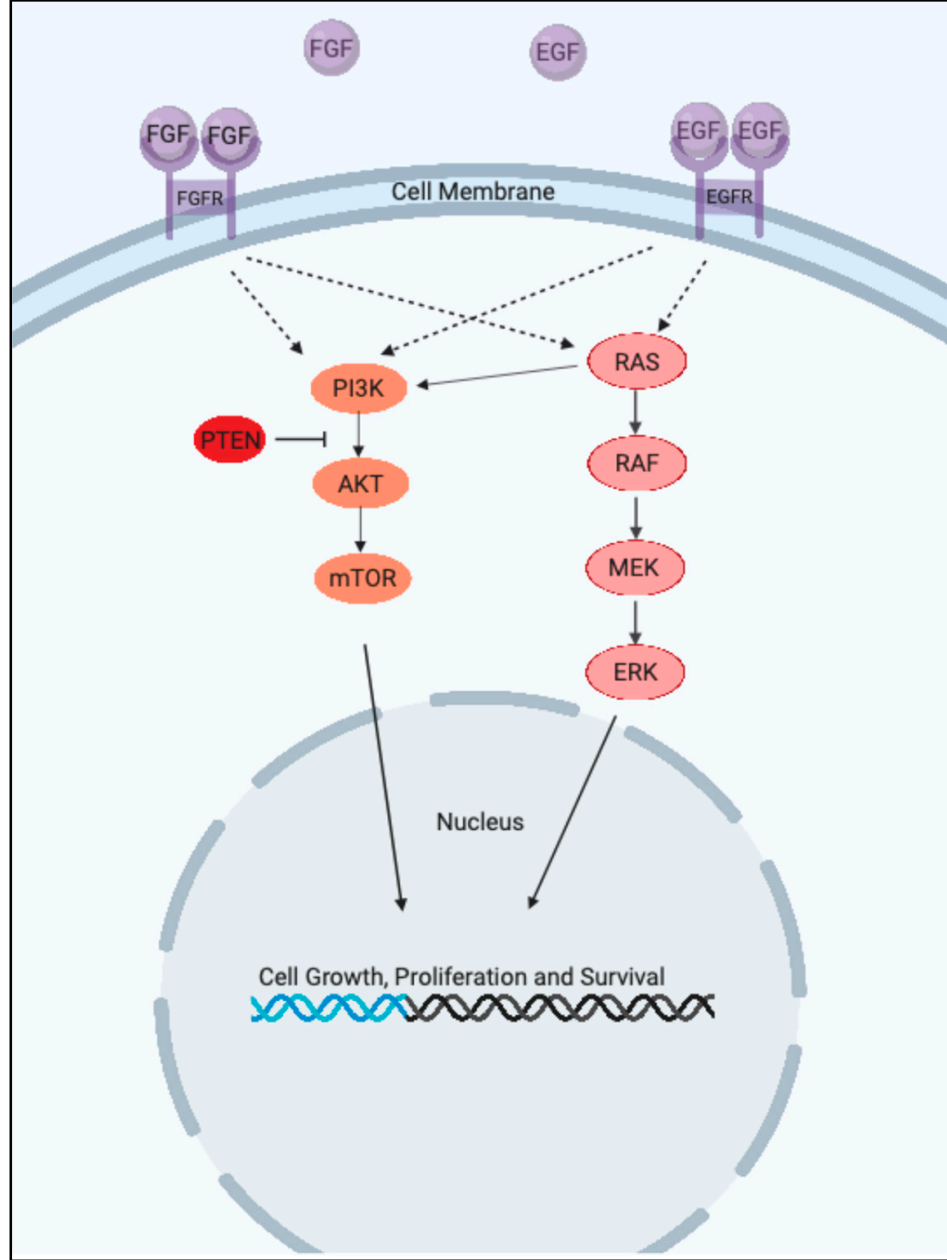
The ERK1/2 & PI3K Pathways. (Created with BioRender.com).

The ERK1/2 & PI3K pathways are the cell’s main signaling pathways for regulating various cellular processes such as cellular proliferation, survival, differentiation, metabolism, and motility. The ERK 1/2 pathway is the main mitogen activated protein kinase (MAPK) pathway and includes the kinase proteins RAS-RAF-MEK-ERK, which successively propagate each other via phosphorylation [1]. The PI3K pathway is similarly a kinase pathway and includes the components PI3K-AKT-mTORC1 [2]. The ERK1/2 & PI3K pathways can be seen in Figure 1 below.

Both pathways receive extracellular signals through a receptor tyrosine kinase (RTK) and when activated transmit a downstream signal to initiate growth-promoting events. As such, both are often overactive in cancer and promote the growth and survival of cancer [3].

Decades of research have centered around attempts to mitigate ERK1/2 and PI3K pathway overactivity in cancer. Such attempts include the development of direct inhibitors for upstream and downstream components, immune system modulation, combination therapies, and more [4,5]. Beyond those relating to efficacy, such therapeutic strategies also raise several questions which are yet to be answered, including those involving drug resistance, toxicity, and use in different cancer types, stages, and alongside possible synergistic partners [6,7].

Resistance to targeted therapies in the ERK1/2 and PI3K pathways also commonly arise through several convergent mechanisms. Common mechanisms include reactivation of RAS signaling, loss of negative regulators such as PTEN, and bypass via upregulation of RTKs, which restore downstream signaling despite inhibition of specific pathway intermediates. Additional resistance arises from activating mutations or amplification of downstream effectors (e.g., ERK1/2), as well as adaptive cellular plasticity that enables tumor cells to circumvent pharmacologic blockade. These mechanisms underscore the need for rational combination strategies and molecular profiling to anticipate and overcome resistance in clinical practice [8,9,10,11,12,13,14].

Additionally, combination therapies targeting both the ERK1/2 and PI3K pathways have emerged as a promising strategy to overcome resistance and enhance antitumor efficacy in cancers with pathway co-activation or compensatory signaling. Preclinical and early clinical studies demonstrate that dual inhibition—such as combining MEK and PI3K inhibitors—can induce synergistic apoptosis and tumor regression, particularly in tumors harboring KRAS, BRAF, or PIK3CA mutations. This approach is increasingly being evaluated in clinical trials, with biomarker-driven patient selection and rational drug combinations showing potential to address the limitations of monotherapy [15,16,17].

In this review, we explore each of the components of the ERK1/2 and PI3K pathways in cancer. We examine the role of each pathway substrate in driving cancer progression and provide an overview of therapeutic strategies which have been developed to combat each component. Finally, we provide a brief summary of future research directions relating to challenges and unanswered questions for each pathway component.

## 2. Growth Factor Receptors

### 2.1. EGFR

The Epidermal growth factor Receptor (EGFR) is an ErbB family transmembrane tyrosine kinase receptor that has a single-chain transmembrane glycoprotein composed of an extracellular ligand binding domain [18]. Ligand activation leads to cellular signaling through multiple pathways, including kinase-dependent and independent mechanisms. This tightly regulated activity controls cellular response to growth factors, influencing autophagy and metabolism [19].

Key activated pathways include phospholipase C, MAPK, extracellular signal-regulated kinases, and Ras GTPase [20]. The MAPK pathway is a critical pathway for signaling from the cell membrane to the nucleus [21]. Ras activation also initiates pathways from the cell surface to the nucleus, interacting with transcription factors to regulate gene expression and cell proliferation [21]. Other downstream signaling pathways EGFR activates include phosphatidylinositol 3-kinase, interleukin 6 transducer, and activator of transcription 3 (STAT3) [22]. Blocking EGFR ligand has been found to decrease CDK activities, leading to cell cycle arrest. This indicates that EGFR is involved in the cell cycle pathway [21]. Additionally, EGFR signaling increases calcium activation by activating PLC and PKC, which influences cell differentiation and proliferation [21]. Once signaling is complete, the EGFR complex is then degraded by a complex of lysosomes and engulfed via endocytosis [23].

EGFR is involved in various cancers, including breast cancer, head and neck squamous cell carcinoma, colon cancer, ovarian cancer, pancreatic cancer, non-small-cell lung cancer (NSCLC), melanoma, and thyroid cancer. In these cancers, EGFR is overexpressed due to various mutations, which leads to an overactivation of EGFR. This leads to increased proliferation, angiogenesis, and resistance to apoptosis, thereby promoting tumor growth and progression [20].

A key feature in cancer progression is the overactivation of the cell cycle mediated by EGFR signaling pathways such as PI3K/Akt/mTOR and RAS/RAF/MEK/ERK. In specific NSCLC patients, an activating mutation in EGFR is found in 10–20% of Caucasians and 50% of Asian patients. This leads to continuous activation of EGFR signaling, which promotes cell proliferation and survival [24]. The specific mutations that continuously activate EGFR include an exon 19 deletion and L858R point mutation. These are known as classical mutations, accounting for 85% of NSCLC caused by EGFR mutations. The remaining 15% are due to rare EGFR mutation, accounting for 30,000 new diagnoses yearly [24]. Rare EGFR mutations include the exon 21 L861Q mutation, EGFR exon 20 insertion mutations, the S768I point mutation, rare EGFR exon 19 mutations, and rare EGFR exon 18 mutations. Among rare EGFR mutations in NSCLC, G719X substitutions are among the more commonly observed mutations [24].

Recent studies have shown that monoclonal antibodies and Tyrosine kinase inhibitors (TKIs) can effectively target the overexpression of EGFR. These treatments work by preventing the dimerization and autophosphorylation of the domain, thus inhibiting downstream signaling through competitive inhibitions and blocking EGFR ligands via monoclonal antibodies [25]. Additionally, successful EGFR inhibition via TKIs is influenced by TP53 gene mutations. Recent findings have shown that cells with TP53 gene mutations show a decreased inhibition of EGFR by TKIs, resulting in a poor prognosis [26].

Cetuximab is an example of a monoclonal antibody used to bind to EGFR. It is also used in combination with irinotecan to treat cancers with overexpression of EGFR. It works by competitively binding the extracellular second L2 domain of EGFR, thus blocking the ligand interaction and downstream signaling. In addition, Cetuximab directs cytotoxic immune effector cells towards the EGFR overexpressed tumor, inhibits the cell cycle at the G1 phase, and induces apoptosis through the expression of the caspase-8 and Bcl-2 proteins [25]. Moreover, Amivantamab is a bispecific monoclonal antibody that targets the EGFR receptor, thereby preventing the activation of downstream signaling pro-growth factors [27].

Furthermore, EGFR TKIs such as Gefitinib, Erlotinib, Afatinib, Dacomitinib, and Osimertinib bind to the tyrosine kinase domain of EGFR, competing with ATP for binding. First-generation EGFR-TKIs, such as Erlotinib and Gefitinib, bind reversibly to the mutant EGFR ATP-binding site. Second-generation inhibitors, like Afatinib and Dacomitinib, bind irreversibly via covalent binding to cysteine residues in the intracellular tyrosine kinase domain, inhibiting autophosphorylation. Third-generation EGFR-TKI, such as Osimertinib and Roclinetib, bind irreversibly via covalent mechanisms to both active and inactive conformations of mutant EGFR, selectively targeting EGFR T790M mutation [22]. By inhibiting EGFR signaling, these TKIs prevent cellular proliferation and survival, hindering the progression of cancer.

Osimertinib—the main EGFR-TKI used in the United States—has been shown to be more effective than first-generation EGFR-TKIs in overcoming drug resistance. It is specifically used against the T790M mutation and is also indicated for tumors harboring exon 19 deletions or exon 21 L858R substitutions [28]. In cases of resistance to Osimertinib, platinum-based chemotherapy is used to intercalate into cancer cell DNA and induce apoptosis [28,29].

Ongoing studies are exploring other approaches to reducing drug resistance. Recent studies indicate that HER3 contributes to drug resistance in EGFR-TKIs in NSCLC due to activation of the PI3K pathway and the overexpression of pro-growth and anti-apoptotic signals via the AKT pathway [30]. As such, Patritumab and lumretuzumab—monoclonal antibodies targeting HER3—are currently being tested in phase I trials [20]. Additionally, Patritumab deruxtecan (HER3-DXd)—a HER3 antibody drug—is also currently undergoing testing in a phase 2 clinical trial for patients with tumor progression after EGFR TKI therapy [28,31]. In addition to HER3, KRAS has been identified as the most frequently mutated oncogene in EGFR-mutated NSCLC, playing a significant role in EGFR-TKI resistance. The KRAS G12C mutant protein inhibitor, a KRAS inhibitor, has shown high efficacy in mitigating drug resistance and slowing cancer progression [20].

Another promising therapy for drug resistance involves angiogenesis inhibitors, which target the signaling of vascular endothelial growth factors. A current phase 3 trial is ongoing with combined therapy of Carboplatin, Paclitaxel, VEGF Bevacizumab, and Atezolizumab, evaluating combined chemotherapy and immunotherapy [30]. Furthermore, ORIENT-31 is a phase III randomized clinical trial evaluating the efficacy of combination therapy with Sintilimab, a PD-1 inhibitor, with or without a Bevacizumab biosimilar, in addition to platinum-based chemotherapy, for patients with advanced EGFR-mutant NSCLC who have progressed after prior EGFR-TKI therapy [30]. Moreover, with the rise in resistance to third-generation EGFR-TKIs, fourth-generation EGFR-TKIs are being tested in clinical trials. These include BLU-945, BBT-176, and JIN-A02 [32]. BLU-945 is being tested for resistance against T790 and C797S mutations. BBT-176 and JIN-A02 are being tested as a novel, fourth-generation TKI for resistance to third-generation EGFR-TKI Osimertinib [28,33].

Furthermore, ongoing studies are now using newer agents such as Mobocertinib and Amivantamab to treat cancers with less common mutations, such as in exon 20, which were previously thought to be undruggable [28]. A recent phase 3 study found Amivantamab combined with Lazertinib to be a better first-line treatment for EGFR-mutated advanced NSCLC than Osimertinib [34]. Another Phase 3 PAPILLON study recently found that Amivantamab treated with platinum-based chemotherapy demonstrated superior efficacy compared to chemotherapy alone in patients with EGFR-mutated NSCLC that has advanced following treatment with Osimertinib. This led the European Commission to approve Amivantamab, in combination with chemotherapy, as the first-line treatment for advanced non-small-cell lung cancer with activating EGFR exon 20 insertion mutations [35].

Lastly, miRNAs are being investigated for their role in mediating resistance to TKIs in NSCLC patients with EGFR mutations. miRNAs have shown effectiveness by activating the PI3K/AKT/mTOR signaling pathway, which regulates upstream factors involved in TKI resistance. Specifically, miR-34a has shown promise in targeting the C-MET gene, known to contribute to EGFR-TKI resistance, and successfully inhibiting the EGFR/PI3K/AKT pathway, thereby reversing Gefitinib resistance in NSCLC cells such as HCC827 (ATCC catalog #CRL-2868, harboring an exon 19 deletion in EGFR) and PC-9 (MilliporeSigma catalog #90071810-1VL, harboring an EGFR exon 19 deletion and often used as a gefitinib-sensitive model) [20,36].

In the past year, fourth-generation EGFR TKIs and novel heterocyclic derivatives have shown improved efficacy against resistant mutations such as C797S, with ongoing trials evaluating their ability to overcome acquired resistance and reduce adverse events [37,38]. Additionally, combination strategies integrating EGFR inhibitors with immune checkpoint blockades are being actively investigated to enhance therapeutic outcomes in resistant and refractory cancers [39,40].

### 2.2. FGFR3

Fibroblast Growth Factor Receptor 3 (FGFR3) belongs to the tyrosine kinase family of receptors, consisting of a transmembrane protein with extracellular, transmembrane, and intracellular domains [41]. The extracellular domain binds Fibroblast Growth Factors (FGFs), initiating a cascade where FGFR3 undergoes dimerization of its intracellular domains and subsequent conformational changes. This triggers transphosphorylation and autophosphorylation of tyrosine residues, activating FGFR3’s tyrosine kinase activity. This activation recruits adaptor proteins to phosphorylated tyrosine residues, initiating downstream signaling pathways like Ras/MAPK and PI3K/Akt. These pathways regulate key cellular processes—including proliferation, differentiation, and survival—by modulating gene expression through activation of transcription factors and signaling cascades that control cell cycle progression and apoptosis [41].

FGFR3 aberrations are implicated in several cancers, including bladder, urothelial, multiple myeloma, endometrial, pancreatic exocrine, and renal cell carcinoma [41]. In bladder cancer, common alterations like FGFR3-TACC3 fusions and gain-of-function mutations (e.g., S249C, R248C) lead to hyperactivation of FGFR3 signaling, promoting tumor growth and progression [42]. Targeting FGFR3 with specific inhibitors such as Small Molecule Tyrosine Kinase Inhibitors (Erdafitinib, Infigratinib) or monoclonal antibodies (Vofatamab) has shown efficacy in inhibiting aberrant signaling pathways like MAPK-ERK and JAK-STAT, thereby suppressing tumor growth [42].

Approximately 20% of patients in multiple myeloma exhibit a translocation event involving t(4;14), resulting in fusion or rearrangement of FGFR3 genes. This genetic alteration leads to constitutive activation of FGFR3 signaling pathways, promoting oncogenesis and disease progression. FGFR3 inhibitors have emerged as promising therapeutic options for these patients, targeting the aberrant kinase activity and downstream signaling cascades [43]. Moreover, fusions of BAIAP2L1 or TACC3 to the 5′ terminal of FGFR3 have been reported in glioblastoma [41]. Current treatments utilize FGFR3 inhibitors to mitigate aberrant activation of the MAPK-ERK and JAK-STAT pathways, thereby enhancing anti-tumor effects [44].

Current treatments for cancers associated with FGFR3 focus on inhibiting its signaling pathways. One approach utilizes First Generation non-selective FGFR kinase inhibitors, which compete with ATP at its binding site and broadly inhibit tyrosine kinases like EGFR, PDGFR, VEGFR, and FLT-3. Examples include Nintedanib, Dovitinib, Ponatinib, Lucitanib, and Derazantinib. More targeted therapies include Second-Generation selective FGFR Kinase Inhibitors such as Erdafitinib and Infigratinib, acting as non-covalent Pan-FGFR inhibitors with broad-spectrum activity against FGFR3. These FGFR3 inhibitors, along with Covalent Pan-FGFR Inhibitors and antibodies, provide confidence in the current treatment options for FGFR3-driven cancers.

Combining multiple drugs has proven effective due to extensive signaling crosstalk between FGFR3 and other pathways, though caution is warranted due to potential increased toxicity [41]. To combat this, many doctors prescribe dual/multi-target inhibitors due to their predictable pharmacokinetics and better patient compliance, encompassing both first and second-generation FGFR TKIs [41]. To counteract drug resistance, FGFR degraders like Proteolysis Targeting Chimeras (PROTAC) are employed, inducing selective intracellular proteolysis to inhibit FGFR signaling pathways effectively [41].

Numerous emerging therapies are aimed at more effectively treating and targeting cancers associated with FGFR3 mutations. In advanced bladder cancer patients who have not responded well to previous treatments, a phase III study is being conducted to evaluate the efficacy of Erdafitinib against chemotherapy or immunotherapy. Additionally, Erdafitinib is the subject of another phase III trial involving advanced cancer patients with FGFR3 genetic alterations who have exhausted standard treatment options [42]. AZD4547, a pan-FGFR inhibitor targeting FGFR3 tyrosine kinases and inhibiting tumor growth, is undergoing clinical trials in patients with multiple cancers featuring FGFR alterations [45].

Several other covalent pan-FGFR inhibitors are in development, such as Pyrazole-benzimidazole CPL304110, which targets FGFR1–4 in bladder, gastric, and squamous cell lung cancers, and KIN-3248, a next-generation pan-FGFR inhibitor showing promising results against mutations resistant to reversible and irreversible FGFR inhibitors [46]. DW14383, another developmental drug, effectively suppresses tumor proliferation and angiogenesis by targeting FGFR1–4 with equal potency [41].

An essential factor in FGFR3 treatment is the potential to resist inhibition, which can occur due to mutations, activation of parallel signaling pathways, or hyperactivation of downstream stimulators. To address this, novel combination therapy approaches are being explored, such as combining Erdafitinib with Midazolam, a cytochrome 450 (CYP) 3A substrate, currently in phase II clinical trials. Other ongoing phase II trials are investigating combinations like Erdafitinib with Midazolam and Metformin, Derazantinib with Atezolizumab, and Futibatinib with Pembrolizumab. Rogaratinib (BAY1163877) and Atezolizumab are also under evaluation in a Phase 1b/2 Study [46].

Additionally, a phase II/III clinical trial comparing Rogaratinib, a novel targeted therapy for FGFR3-driven cancers, with chemotherapy has shown comparable efficacy and a favorable safety profile in patients with FGFR3 genetic abnormalities [47]. Another promising agent, E7090, a reversible FGFR3 inhibitor, is currently undergoing phase II trials demonstrating efficacy in FGFR3 inhibition. Furthermore, Debio 1347, an ATP competitive inhibitor targeting FGFR1-3, is also in phase II trials, highlighting ongoing efforts to enhance treatment options for FGFR3-driven cancers [44]. Moreover, monoclonal antibodies such as Vofatamab or B-701 have demonstrated efficacy in phase II trials when combined with Docetaxel or Pembrolizumab. Vofatamab functions by inhibiting dimerization through binding to the extracellular domain of FGFR3 [44]. Neutralizing antibodies and FGF ligand traps, like Vofatamab or B-701, have shown promise by blocking ligand-receptor interactions, thereby disrupting FGFR3 signaling [46].

Finally, next-generation FGFR inhibitors such as Lirafugratinib and LOXO-435, as well as the FGFR2-specific antibody Bemarituzumab, are in clinical development and demonstrate reduced risk of hyperphosphataemia and activity against resistance mutations [48]. There is also increasing interest in expanding FGFR inhibitor indications and combining these agents with immunotherapies to address bypass signaling and isoform switching in resistant tumors.

In addition to the search for new treatments, recent literature has explored improved methods for diagnosing FGFR3 mutations. Liquid biopsy represents a novel alternative to traditional tissue biopsies, facilitating early diagnosis and detection of gain-of-function mutations in FGFR3 [42].

## 3. ERK1/2 Pathway

### 3.1. RAS

The RAS protein family is a group of 39 protein GTPases that play a critical role in various cell processes, including cell differentiation, migration, adhesion, proliferation, and survival [49]. They are membrane-bound signaling proteins that are constantly switching between a GDP-bound (inactive) and GTP-bound (active) state with the help of Guanine exchange factors (GEFs) and GTPase-activating proteins (GAPs) [49,50]. The active RAS-GTP complex can bind to several different effector proteins, including Raf, PI3K, and RalGDS, causing downstream signaling and action in the MAP kinase, Akt/mTOR, and Ral pathways, respectively [50]. The G domain of RAS is meant to bind these effectors to produce the necessary downstream signals, while the C terminal plays a role in binding to the cell membrane [51]. Due to RAS’s nature as an oncogene and signaler of downstream action, mutations in RAS can lead to a state of constant activity, potentially contributing to the development of cancer [51].

Ras is the most frequently mutated oncogene in cancer, occurring in about 19% of all cancers, with KRAS being the most commonly mutated isoform [50,51]. The KRAS isoform most commonly causes pancreatic, colonic, biliary tract, and lung cancers [50]. NRas mutations frequently develop into cancers such as malignant melanoma and acute myeloid leukemia, while HRas mutations lead to squamous cell carcinoma and transitional cell carcinoma [50]. The common pathogenesis of the RAS mutation is a single point missense mutation in either the G12, G13, or Q61 codons that bring about the disabling of the intrinsic GTPase activity or GAP-binding ability of RAS, leading to a constitutively active RAS-GTP bound state that causes downstream signaling in the Raf, PI3K, and RaGDS pathways, giving rise to cellular transformation and tumorigenesis [49,52,53].

Inhibiting the effects of the RAS protein can involve a series of different approaches, including targeting upstream proteins, downstream proteins, RAS itself, as well as RNA interference [51]. Tipifarnib, an FTase inhibitor, targets the upstream proteins farnesyltransferase (FTase) and geranylgeranyltransferase (GGTase), which both play critical roles in RAS translocation to the cell membrane, a necessary step for RAS protein biological activity [51]. Though it has shown to be efficacious in inhibiting tumorigenesis to target both FTases and GGTases, the toxicity associated with this dual-inhibitory mechanism limits the benefit of the drug [51]. Another drug that interferes with the membrane-binding mechanism of RAS is Salirasib, which competitively inhibits RAS by competing for membrane binding sites [49]. Recent trials with Salirasib have demonstrated positive results against advanced solid tumors with a subset of KRAS mutations [49]. Palmitoylation is a necessary step for RAS-membrane interactions in H-RAS and N-RAS isoforms. Therefore, Depalmitoylation inhibitors and Palmitoyl acyltransferases have been tested and shown to have activity against RAS, but the questionable adverse effects that come with these drugs have halted further clinical development [49].

Inhibition of downstream pathways such as the ERK and PI3K signaling pathways could mitigate the effects of continuous RAS activation [49]. One such mechanism that these pathways could be inhibited is via inhibition of the RAS-RAF-MEK-ERK cascade through RAF kinase inhibitors, ERK inhibitors, or MEK inhibitors, all of which can play a role in terminating the transduction of growth signals to the cell nucleus. Vemurafenib and Dabrafenib are two RAF inhibitors that act on RAF monomers, effectively inactivating the RAF protein from continuing the cascade [49]. However, although these two drugs inhibit RAF kinase, they have been shown to simultaneously activate the downstream ERK signaling pathway, and have not been effective in treatment against RAS-mutated cancer [49]. Belvarafenib and LXH-254, two pan-RAF inhibitors, target both the RAF monomer and dimer isoform, and are undergoing clinical evaluation for RAS-mutant advanced solid tumors [49].

MEK inhibitors allosterically inhibit the MEK protein by binding to a site near the ATP binding domain of MEK. Trametinib, Selumetinib, Cobimetinib, and Binimetinib have been approved for treatment of patients with BrAF V600E/K melanoma, but tumor inhibition in RAS-mutated cancers have not been successful due to their rapid emergence of resistance. Therefore, instead of being used as a monotherapy, these MEK inhibitors have been combined in treatment with conventional chemotherapeutic agents, systemic immunotherapies, and Raf inhibitors. This approach has demonstrated preclinical efficacy in KRAS/p53-mutant lung cancer, creating optimism for further development of combinatorial treatments that can be used for RAS-driven cancers [53].

ERK inhibitors such as LY3214996 work by competing for the ATP-binding spot on ERK. While it has exhibited effective anti-tumor activity, the therapeutic index of this inhibitor is limited, thereby limiting its ability to act as a monotherapeutic agent. LY3214996 was found to be well-tolerated and worked well in combined modality settings in xenograft models of KRAS-mutant NSCLC and colorectal cancer, and has therefore been advanced into human clinical trials in cancers of RAS mutations [53].

Although formerly considered impossible, directly targeting RAS proteins has emerged as a mechanism to treat RAS-driven cancers. A recent discovery in the crystal structure of RAS has allowed for a strategy that inhibits the SOS-RAS interaction, effectively keeping RAS in its inactivated state. DACI, BAY-293, and BI 1701963, have all demonstrated their ability to effectively inhibit this reaction while working either as a monotherapy or as a combinatorial therapeutic [51].

### 3.2. RAF

The RAF protein family is composed of three serine/threonine kinases (ARAF, BRAF, CRAF) [54] that regulate various cellular processes such as cell cycle progression, proliferation, metabolism, migration, differentiation, and apoptosis [4]. RAF proteins have a RAS-binding domain, which it uses to become activated from its initially inactive cytosolic monomer form [54]. The RAS-RAF interaction results in the recruitment of RAF to the cell membrane and the formation of RAF dimers [55]. The newly formed dimers act as kinases to phosphorylate MEK, activating a phosphorylation cascade that results in the expression of transcription factors that lead to the transcription of cell proliferation genes [55]. As RAF is responsible for a variety of downstream effects, its mutation and dysregulation can be found in cancer-generating pathways [4].

Mutations in RAF are quite prevalent in human cancer (BRAF mutations are present in about 6% of human cancers) [54], and are responsible for many different types of cancer, including colorectal cancer [56], melanoma, breast cancer, ovarian cancer, thyroid cancer, and prostate cancer [4]. More specifically, the BRAF mutation is quite common in these cancers as well, accounting for 30% of ovarian tumors, 40% of papillary thyroid cancers, 50–70% of melanomas, and nearly 100% of hairy cell leukemias [55]. The most common activating BRAF mutation in BRAF-associated cancer is the substitution of valine with glutamic acid at the codon 600 (V600E) [57]. This mutation is so heavily associated with cancer because it allows BRAF to be activated independently of RAS, leading to constant BRAF activation and therefore constant activation of the RAS-RAF-MEK-ERK phosphorylation cascade [57]. Recognizing this mutation’s responsibility for the various cancers that mutant RAF is involved in, several current and emerging therapeutics for RAF-involved cancers have targeted this pathway.

The treatments against RAF-mutated cancers mainly target proteins either upstream of RAF, downstream of RAF, or RAF itself. BRAF inhibitors such as Vemurafenib and Dabrafenib are ATP competitive inhibitors of mutant BRAF V600E [58]. Therefore, they directly act on the mutated BRAF V600E and act as strong inhibitors of RAS and MAPK in tumors that expressed the BRAF V600E mutant [54]. Due to the rapid adaptive resistance that is gained by these cancers, the BRAF inhibitors are commonly paired with MEK inhibitors as a combination therapy. This combination therapy had a higher response rate, higher progression free rates and higher rates of overall survival [54]. Cetuximab and Panitumumab are monoclonal antibodies that target the EGFR receptor. In a study performed in 2017 that dealt with 48 mutant BRAF profile patients, 53% of patients receiving Cetuximab had early tumor shrinkage, compared to 33% receiving Bevacizumab [55].

Bevacizumab is an agent that stops tumor angiogenesis and growth by inhibiting the interaction between circulating Vascular Endothelial Growth Factor (VEGF) and its receptor [55]. Several studies regarding patients with colorectal cancer with BRAF mutation have found Bevacizumab therapy with chemotherapy to be quite successful, improving overall survival compared to patients just undergoing chemotherapy alone [55].

Another way to inhibit the BRAF-mutated cancer is limiting the downstream components of the pathway. Selumetinib is a strong MEK inhibitor that has shown efficacy in BRAF V600E thyroid cancer [59]. While it can be effective, dose reduction and complete discontinuation of the drug was reported due to the severe side effects of fatigue, diarrhea, and rash that it may cause [59].

Sorafenib is a drug that has been approved for patients with advanced, iodine-refractory thyroid cancer [58]. Sorafenib is a multikinase inhibitor that has many different targets, including both the wild-type and mutant BRAF V600E [58]. In a study performed analyzing the effectiveness of the drug, Sorafenib was found to increase the Progression Free Survival (PFS) of patients that were taking it, and a larger increase in PFS was specifically seen in patients with a BRAF V600E mutation [58].

Increasing the immune system’s capabilities is another approach that can be used to tackle RAF mutant cancer. Normally, tumors express immune checkpoint proteins to suppress the immune system’s response to cancer. Two examples of therapies that block these checkpoint inhibitor proteins are Nivolumab and Ipilimumab, which are monoclonal antibodies against the inhibitor proteins that can reverse tumor-induced immunosuppression [55].

Elevated MEK activity is very common in RAF induced malignancies [4]. Therefore, targeting the downstream MEK proteins is a method in which the effects of the cancer can be mitigated. Trametinib, which is a MEK inhibitor, was found to be effective against BRAF V600E-mutated metastatic melanoma. Additionally, Selumetinib, which is a highly specific MEK inhibitor, was approved to be used for the treatment of children with neurofibromatosis type 1 [4].

In patients that have tumors that have developed resistance to the other drugs mentioned, an alternative pathway inhibition that could be used is inhibiting the PI3K/AKT pathway. Although this will not work for many of the mutant RAF tumors, a small portion of these tumors rely on the compensatory PI3K/AKT signaling cascade. PI3K inhibitors like Alpelisib have shown promising results when used in combination with Vemurafenib. Additionally, in more challenging BRAF-mutated colorectal cancers, a combinatorial therapy of Alpelisib, Encorafenib, and Cetuximab has shown to be effective as well [4].

### 3.3. MEK

Mitogen-activated extracellular signal-regulated kinase (MEK) is a protein kinase in the well-identified Ras pathway. MEK activation begins through the binding of growth factors such as EGFR, activating a receptor tyrosine kinase, leading to receptor dimerization and phosphorylation and the activation of Ras. Ras then activates a kinase cascade mainly with B-Raf. B-Raf phosphorylates MEK1/2, receptor tyrosine, and serine/threonine kinases which go on to phosphorylate and activate ERK1/2. Current research suggests that MEK specifically activates ERK1/2, serine/threonine kinases that promote phosphorylation of pRB and downregulation of key cell cycle regulators such as p15, p16, and p21, along with decreased expression of pro-apoptotic BCL-2 family proteins. Additional processes involved in cell proliferation and differentiation through MEK1/2’s activation of ERK1/2 include the activation of multiple growth transcription factors, the upregulation of telomerase through hTERT, increased expression of Epithelial-to-Mesenchymal proteins, as well as the induction of angiogenesis, and advanced antigen hiding processes [60].

Gain-of-function mutations, generally upstream of MEK1/2 in the MAPK pathway, can lead to multiple subsets of tumors [60]. MEK inhibitors, when paired with direct therapy, have proven to synergistically increase the apoptotic effect as well as stall cancer-cell resistance [61]. Gain-of-function mutations in EGFR can lead to increased activation of the MAPK pathway and the subsequent development of NSCLC. Treatment of NSCLC with EGFR TKIs such as Osimertinib has been effective until cancer cells acquire resistance to the therapy. A study regarding Osimertinib treatment found that while Osimertinib initially reduced levels of phosphorylated ERK1/2, phosphorylated ERK1/2/s inhibition would decrease, and in certain instances even increase, with time. However, when Osimertinib was combined with Trametinib (MEK inhibitor), it displayed significantly enhanced potency, as evidenced by enhanced cleavage of caspase 3 and PARP (apoptotic markers) in EGFR-mutant NSCLC cells. These findings suggest that combining Osimertinib with MEK inhibition can potentially delay cell resistance and enhance the induction of apoptosis in future treatment of EGFR-mutant NSCLC [62].

Similarly, driver mutations in the BRAF gene can form NSCLC, anaplastic thyroid cancer, and melanoma [63]. BRAF inhibitors are effective in treatment but were limited by acquired resistance in cancer cells; when BRAF inhibitor treatment was paired with targeted MEK inhibition, this acquired resistance was effectively delayed [64]. In one study regarding the treatment of BRAF-induced metastatic melanoma, patients treated with Dabrafenib plus Trametinib (BRAF therapy + MEK inhibitor) had reduced their risk of death by 31% when compared to Vemurafenib alone (BRAF inhibitor), and prolonged free survival was significantly higher with the combined therapy when compared to Dabrafenib plus placebo [63]. Similar results were demonstrated regarding the Vemurafenib + Cobimetinib (BRAF therapy + MEK inhibitor) combined treatment of BRAF-induced craniopharyngioma, which found that of 16 participants, 15 had a medium tumor reduction of 83% [61]. Additionally, a study regarding the long-term outcomes of BRAF/MEK in treatment in melanoma patients determined more than 75% of patients who experienced progression-free survival of 4 years from BRAF/MEK inhibitors continued to exhibit lasting and effective anti-tumor responses even after an average follow-up period of nearly 8 years, underscoring the frequent positive outcomes seen in patients who derive prolonged benefit from BRAF/MEK inhibitors [65].

Pancreatic tumors carry the highest incidence of a KRAS mutation at approximately 70–90% [60], and reciprocally activated RAS/MEK pathways have conferred therapeutic resistance in pancreatic ductal adenocarcinoma. Treatment of these pancreatic KRAS tumors has proven especially difficult due to dense desmoplastic stroma which denies effector immune cells or therapeutics access to the tumor, creating an immunosuppressive microenvironment and tumor that is invulnerable to immune checkpoint inhibition. While previous studies have confirmed combined MEK and STAT3 inhibition effective in overcoming therapeutic resistance and altering stromal architecture, a present study has determined that MEK/STAT therapy plays an active role in bypassing immunotherapy resistance in PDAC as well, decreasing stromal inflammation and increasing cancer-associated fibroblasts with mesenchymal properties [66]. The combination of MEK and STAT3 inhibition shows promise in improving treatment outcomes in pancreatic ductal adenocarcinoma.

MEK inhibitors are associated with a high frequency of mucocutaneous toxicities (rash, xerosis, dermatitis, paronychia), gastrointestinal events (diarrhea, nausea, colitis), ocular toxicities (blurred vision, MEK inhibitor-associated retinopathy), and cardiovascular effects (hypertension, decreased ejection fraction, QTc prolongation), with most adverse events being grade 1–2 but grade ≥ 3 events requiring dose reduction or interruption in a significant minority of patients. Dose-limiting toxicities include severe rash, colitis, retinopathy, and cardiac dysfunction [67,68,69,70,71,72,73,74,75]. Management strategies emphasize early recognition, patient education, and multidisciplinary supportive care: dermatologic events are managed with topical corticosteroids and emollients, gastrointestinal toxicities with antidiarrheals and, if severe, drug interruption, ocular events with prompt ophthalmologic evaluation and possible dose modification, and cardiac events with regular monitoring and cardiology input. Most toxicities are reversible with dose adjustment or temporary discontinuation, but rare severe events (e.g., gastrointestinal perforation, sight-threatening retinopathy, or heart failure) may require permanent cessation.

### 3.4. ERK

Extracellular signal-related kinase (ERK) is a protein kinase in the RAS pathway. MEK1/2 are protein kinases that phosphorylate and activate ERK1/2; ERK1/2 then phosphorylates over 70 different substrates possessing nuclear transcription factors. Through this mechanism, the ERK pathway plays a pivotal role in cellular proliferation, survival, and differentiation, making it an attractive target for intervention in various cancers where dysregulation of this pathway is common. By inhibiting ERK activation, these inhibitors can disrupt aberrant signaling cascades, leading to the suppression of tumor growth and metastasis. Resistance to MAPK inhibitors often involves abnormal ERK regulation and increased ERK phosphorylation as well. Utilization of an ERK1/2 kinase inhibitor could potentially overcome MAPK inhibitor resistance in tumor cells. While several drugs targeting ERK have been developed, most remain in preclinical trials, demonstrating an untapped potential for the development of ERK inhibitors in cancer therapy [76].

The overexpression of HER2, an oncogene common in many forms of cancer including breast cancer, has been associated with the abnormal activation of ERK [76]. Additionally, previous studies have determined that there is a compensatory increase in activation of the HER2/ERK signaling pathway in response to PI3K/Akt/mTOR inhibition in the treatment of breast cancer [77]. MicroRNAs, a biological agent and form of cancer treatment, work by binding and inducing the degradation of mRNA for various proteins following the process of DNA transcription. MicroRNA 543, a recently discovered microRNA, was found to be expressed 2.87 times less in breast cancer tissue when compared to normal tissue. In a study investigating the role of miR-543 in the regulation of ERK, it was determined through both Western blot and a dual-luciferase experiment that miR-543 directly targets ERK for degradation [78]. Similarly, Bozepinib, an anti-tumor medication, also targets the HER2/ERK pathway, as can be evidenced on Western blot through the complete inhibition of phosphorylated HER2 following treatment with 5 μM of Bozepinib after 2 h on breast cancer cells [79]. These results shed light on a potential mechanism for modulating ERK activity and the development of novel therapeutic strategies in the treatment of breast cancer.

Erk inhibitors can play a critical role in the mediation of acquired cancer-cell resistance as well. Osimertinib, a third-generation EGFR inhibitor, has proven effective in the treatment of EGFR-mutated NSCLC as shown through a decreased level of p-ERK following treatment with Osimertinib. However, as treatment with Osimertinib progresses (24–48 h), p-ERK levels in these cells rebound to their original values or even elevate beyond their baseline, demonstrating an acquired resistance to Osimertinib therapy. When Osimertinib was paired with VRT752271, an ERK inhibitor, the combination therapy displayed a much more potent effect in decreasing the survival of EGFR-mutant NSCLC cell lines [62]. This combination therapy offers a new approach to overcoming acquired resistance in EGFR-mutated NSCLC.

While one pathophysiology that results in NSCLC has been related to EGFR mutation and increased ERK activation, aberrant ERK activation can lead to NSCLC through other avenues as well. Cyclooxygenase 2, a rate-limiting enzyme in the formation of prostaglandins, has been overexpressed in a multitude of cancers. Prostaglandin E2, a prostaglandin produced by COX2, has been shown to significantly increase phosphorylated ERK levels in starved NSCLC cell lines; this positive relationship between PGE2 and ERK was further confirmed by dose-titration experiments which demonstrated increased p-ERK levels following increased PGE2 concentrations. Studies have also demonstrated that PGE2-responsive cells were found to maintain elevated p-ERK levels when treated with EGFR inhibitors, results which attest to the role PGE2 may play in EGFR inhibitor resistance [80]. Future treatments for NSCLC may benefit from targeting the interplay between COX2, PGE2, and ERK, to overcome EGFR inhibitor resistance and improve therapeutic outcomes.

Additional ERK therapies include Ulixertinib and LY3214996, both of which work by competitively inhibiting ERK1/2. Ulixertinib is currently undergoing phase I and phase II clinical trials in the treatment of gastrointestinal malignancies when paired with hydroxychloroquine. Similarly, LY3214996 has demonstrated notable anti-tumor activity both as a singular treatment and as a combined therapy with Hydroxychloroquine. Future phase II clinical trials of LY3214996 both as a monotherapy and when paired with Hydrochloroquine may provide promising results in the treatment of late pancreatic cancer [81]. Vx-11e, a selective inhibitor of ERK2, has been shown to promote intrinsic apoptosis in cancer cells by enhancing reactive oxygen species (ROS) production, which leads to mitochondrial dysfunction and the subsequent release of pro-apoptotic factors such as cytochrome c. This ROS-mediated mitochondrial pathway amplifies apoptotic signaling, distinguishing it from extrinsic apoptosis pathways. In addition to its pro-apoptotic effects, studies have also linked Vx-11e to regulation of autophagy via MAPK signaling, highlighting its broader role in cell death pathways. Due to these anticancer activities, Vx-11e is currently being considered as a treatment for osteosarcoma [81,82].

ERK inhibitors, including agents such as Ulixertinib, LY3214996, and AZD0364, are associated with a toxicity profile characterized primarily by dermatologic adverse events (acneiform and maculopapular rash, pruritus), gastrointestinal symptoms (diarrhea, nausea), and neurologic effects (fatigue, peripheral neuropathy), with dose-limiting toxicities most frequently related to grade ≥ 3 rash, neurotoxicity, and, less commonly, retinal vein occlusion or visual disturbances [83,84,85,86,87,88]. Dermatologic toxicities occur in up to 79% of patients and are managed with topical corticosteroids, oral antihistamines, and dose modification as needed. Neurotoxicity and peripheral neuropathy, as observed with CC-90003, may necessitate drug discontinuation and require close monitoring. Gastrointestinal events are typically mild to moderate and managed with supportive care, but severe cases may require dose reduction or interruption. Intermittent dosing schedules and combination strategies (e.g., with PI3K/mTOR or CDK4/6 inhibitors) are under investigation to mitigate toxicity while maintaining efficacy. Early recognition, patient education, and multidisciplinary supportive care are essential for optimal management and to preserve dose intensity.

## 4. PI3K Pathway

### 4.1. PI3K

Phosphoinositide-3-kinase (PI3K) is involved in signal transduction and plays an important role in regulating cell growth, proliferation, survival, and angiogenesis in mammalian cells. Class I PI3Ks are heterodimers composed of a regulatory subunit—most commonly p85α, p85β, or the shorter splice variant p55—and a catalytic subunit, typically one of the p110 isoforms (p110α, p110β, p110δ, or p110γ) [6]. When PI3K is stimulated by growth factors and cytokines [6], PI3K catalyzes the synthesis of second messenger phosphatidylinositol (3,4,5)-trisphosphate (PIP3) using the catalytic subunit p110 [89]. In turn, PIP3 recruits phosphoinositide-dependent kinase-1 (PDK1) and protein kinase B (AKT) to the plasma membrane, where PDK1 phosphorylates AKT at Thr308 and the mTORC2 complex phosphorylates AKT at Ser473, together resulting in full AKT activation. The activation of AKT second to PI3K activation leads to a signaling cascade which activates the mammalian target of rapamycin (mTOR). The activated mTOR upregulates processes stimulating cell growth such as transcription, translation, protein synthesis and cell cycle progression [90].

The activation of the PI3K signaling pathway plays a significant role in cancer development [91]. Specifically, a frequent mutation in PI3K associated cancers involves the PIK3CA gene. These gain-of-function mutations involve the kinase and helical domains of p110α [89]. p110α is important to cellular proliferation because it is involved in vascular remodeling [92]. Thus, a mutation in the PI3K pathway causes hyperactivation and the continuation of growth signals in a cancerous cell, resulting in tumor proliferation. This is the most commonly mutated oncogene in many tumor lineages, such as colorectal, lung, gastric, prostate and cervical cancers [91]. This mutation is also frequently found in breast adenocarcinoma and head/neck cancers [89]. For example, mutations in the p110α subunit (PIK3CA) have been found in around 40% of HR+/HER2− or HER2+ advanced breast cancer tumors [90]. Since over-expression of PI3K is found in a vast array of cancers, there has been a major push towards the development of PI3K inhibitors [89].

There are a number of PI3K inhibitors currently used as therapeutic agents for cancer, as the hyperactivity of PI3K is significantly correlated with tumor progression, angiogenesis, and cancer cell invasiveness. Drugs that target PI3K are divided into three major classes: pan-PI3K inhibitors, isoform-specific inhibitors, and dual PI3K/mTOR inhibitors [6]. Although Pan-PI3K inhibitors target all four isoforms of class I PI3K, they pose an increased risk to the development of metabolic-related malignancies [92]. Copanlisib was approved in May 2017 as a pan-class I PI3K inhibitor used to treat adults with follicular lymphoma [92]. Current studies are exploring the efficacy of Copanlisib in combination with Trastuzumab to treat HER2+ breast cancer [90]. Although Copanlisib has a low incidence of sever toxicities, some side effects include hyperglycemia, diarrhea, and hypertension.

Taselisib and Duvelisib are two other pan-PI3K inhibitors that have been used to treat cancer, however Taselisib has been negated due to overwhelming systemic toxicity [89,92]. There are four isoforms of PI3Ks—(α, β, δ, γ)—and isoform-specific PI3K inhibitors target each of the forms individually. A powerful PI3Kα inhibitor is Alpelisib, a drug that has been approved in May 2019 for the treatment of postmenopausal women, and men, with HR-positive, HER2-negative, PIK3CA-mutated, advanced or metastatic breast cancer [92,93]. Alpelisib was very effective in clinical trials when combined with Fulverstrant and currently, other combination therapies are in development [92]. Toxicity of hyperglycemia should be noted [89]. Recently, PI3Kβ inhibitors include AZD8186, GSK2636771 and PI3Kδ inhibitors include Idelalisib Duvelisib, Umbralisib and PI3Kγ inhibitors IPI-549. Unfortunately, toxicity is associated with these therapeutic medications, and such adverse effects include hyperglycemia, cutaneous reactions, diarrhea, hepatotoxicity, hypertension and pneumonitis [92]. Lastly, several dual PI3K/mTOR inhibitors are currently in Phase I/II clinical trials. For example, clinical studies regarding the effect of Dactolisib (NVP-BEZ235) on patients with relapsed or refractory acute leukemia and patients with metastatic breast cancer are ongoing [92].

Recent clinical trials have focused on subtype-selective PI3K and AKT inhibitors, including the development of PROTAC degraders, which aim to minimize toxicity and overcome resistance associated with PTEN loss and compensatory pathway activation [94]. Combination regimens targeting PI3K/AKT alongside other pathways, such as ERK or CDK4/6, are being evaluated to improve efficacy in biomarker-selected patient populations.

Although PI3K inhibitors target breast cancer, their efficacy is limited by tumor toxicity. Going forward, methods should be developed to mitigate the occurrence of hyperinsulinemia and hyperglycemia, as they reduce to full potential of the drugs. Dose and scheduling could be a solution to improving the therapeutic index. In addition, consideration should be given to patient stratification [89]. Multiple phase I, II, and III clinical trials are currently ongoing evaluating the role of PI3K inhibitors. For example, Buparlisib is in multiple clinical trials in combination with either Fulvestrant or placebo in postmenopausal ER+/HER2− metastatic breast cancer. In addition, combination of PARP inhibitors and the combination of endocrine therapy, PI3K inhibitors and CDK 4/6 inhibitors are currently under investigation [95].

PI3K inhibitors exhibit frequent, dose-limiting toxicities including hyperglycemia (especially with PI3K-selective agents), rash, diarrhea/colitis, stomatitis, hepatotoxicity, and infections, with severity and spectrum varying by isoform selectivity [92,96,97,98,99,100,101]. Hyperglycemia and gastrointestinal events are the most common reasons for dose reduction or discontinuation, while rare but serious events such as Stevens-Johnson syndrome, febrile neutropenia, and intestinal perforation have been reported. Management strategies emphasize early recognition, patient education, and proactive monitoring; supportive care includes metformin for hyperglycemia, dexamethasone mouthwash for stomatitis, antidiarrheals and corticosteroids for colitis, and temporary drug interruption for severe toxicity. Intermittent dosing, combination regimens, and next-generation isoform-selective agents are under investigation to improve tolerability.

### 4.2. AKT

AKT is a kinase that mediates many cellular functions such as cell growth, proliferation, protein synthesis, glucose metabolism, and the inhibition of apoptosis through BAD and BCL-2 like protein 11 [102]. In addition, AKT inactivates FOXO through phosphorylation, which is a transcription factor involved in the expression of pro-apoptotic genes. AKT also downregulates p53 through protein degradation and activates mTOR kinase, which promotes cell proliferation [103]. The binding of ligands including growth factors such as IGF-1, PDGF, cytokines, hormones, and mitogens to cell membrane receptors triggers AKT activation. When a growth factor binds to a receptor tyrosine kinase, it targets the regulatory subunit of PI3K (p85), which in turn activates the catalytic domain of PI3K (p110). This sequence activates AKT, which undergoes a conformational change, phosphorylates and modulates several substrates that play a role in carcinogenesis [102]. Interestingly, activation of PI3K simultaneously results in activation of AKT in all cells and tissues [104].

There are three isoforms of AKT, all associated with cellular function. AKT 1 is important for cell survival, AKT 2 plays a role in glucose homeostasis [105] and AKT 3 is critical for brain development [102]. Amplification of the AKT1 isoform is more commonly associated with cancers such as breast, lunch, ovarian, pancreatic and gastric carcinomas [102]. Increased AKT1 activity has been observed in around 40% of breast and ovarian cancers and in over 50% of prostate cancers. Furthermore, the overactivation of AKT2 has also been documented in about 30–40 percent of ovarian and pancreatic cancers, while AKT3 may contribute to the level of aggressiveness of hormone independent cancers [106].

There are multiple therapeutic agents that act as AKT inhibitors and block the PI3K/AKT pathway, whose overexpression might stimulate cancer growth. AZD5363 is an oral ATP-competitive pan-AKT inhibitor whose results in clinical trials showed effectiveness against tumors with the AKT1^E17K^ mutations. GDC-0068 (Ipatasertib) is another oral ATP-competitive pan-AKT inhibitor that blocks AKT signaling in a number of cancers, including ovarian, prostate, breast, glioblastoma, colorectal, NSCLC, and melanoma. A phase 1 study of Ipatasertib showed the drug successfully and safely targeting AKT in solid tumors. This drug is now being investigated further in phase II studies [106]. A previous study compared Ipatasertib + Paclitaxel with a placebo + Paclitaxel in 124 triple-negative breast cancer patients and found prolonged overall survival in the Ipatasertib + Paclitaxel group. The pairing of Ipatasertib with a non taxane based chemotherapy is currently in a phase II PATHFINDER trial [107]. Although not enrolled in clinical trials yet, AT7867 and CCT128930 exhibited anti-tumor activities against glioblastoma in xenografts. This leads to the potential of possible human clinical trials. GSK2110183 is an ATP-competitive AKT inhibitor that aims to attenuate the levels of AKT substrates in certain BT-474 breast cancer and LNCaP prostate cancer lines. Results are not publicly available [106].

Furthermore, there are new compounds that are AKT-inhibitor targets that are currently being developed. An AKT inhibitor Borussertib has shown preclinical activity in cell lines and Capivasertib was recently tested in oral cancer cell lines. Many emerging therapies look at the efficacy of AKT inhibitors when paired with other biological agents targeting alternative pathways of carcinogenesis. IPATunity150 is a trial open to accrual that will compare Ipatasertib + Fulvestrant + Palbociclib and placebo + Fulvestrant + Palbociclib in a population of endocrine-resistant HR positive breast cancer. There are also many studies in the works that associate AKT inhibitors with immunotherapy. For example, a current study aims to see the efficacy of Ipatasertib and Atezolizumab in certain cancers. Finally, there is an ongoing phase I trial that tests the efficacy of administeringCapivasertib, Durvalumab, and Olaparib together [107].

### 4.3. mTOR

The mammalian target of rapamycin, mTOR, is a serine/threonine kinase which functions through two distinct complexes, mTOR complex 1 (mTORC1) and mTOR complex 2 (mTORC2) [108]. Both of these complexes play critical roles as they modulate both processes that halt cell growth and proliferation such as autophagy, while also regulating pro-growth factors such as the synthesis of organic molecules [108]. The pro-growth pathway is stimulated by growth factors that are capable of activating both mTORC1 and mTORC2. Mechanistically, this occurs as growth factors lead to stimulation of mTORC2 activating the protein families AKT and AGC, ultimately leading to increased cell survival [109]. In addition to being a downstream target, AKT is also believed to be upstream of mTORC2. This dynamic creates a feedback loop in which mTOR2 can fully activate AKT leading to increased cell growth stimulation. PI3K has also been shown to be an upstream inducer of mTORC2. Due to this and mTORC1’s negative feedback loop with insulin/PI3K signaling this ultimately means mTORC1 can regulate mTORC2 [108]. Given this, the modulation of mTORC1 is essential given its influence on cellular processes. mTORC1 is inhibited when the cell undergoes stressful conditions such as decreased glucose, ATP, or oxygen levels leading to decreased cellular metabolism [108]. This may provide a mechanism for how mutated mTORC1 may lead to tumorigenesis. Given mTOR’s role in both cell destruction and growth pathways, its involvement in cancer formation has been discussed in great detail in several prior reviews [108,109,110,111].

mTOR plays a role in the formation of some drug-resistant cancers in digestive tract, respiratory, kidney, and skin [110]. In part, this is due to mTOR pathways increasing in activity in response to several classes of drugs, such as tyrosine kinase inhibitors and BRAF inhibitors [110]. When looking at gastric cancer cell lines, one study found greater than half had overexpression of mTOR in addition to overexpression of several upstream regulators [112]. Colorectal cancers have similar findings with both mTORC1 and mTORC2 demonstrating overexpression [110]. In bladder cancer as well as primary prostate tumors there are mTOR pathway mutations in 40% of cases. Both loss of regulators such as phosphatase and tensin homolog (PTEN, located on chromosome 10), as well as amplification of mTOR activators such as AKT, can lead to unchecked PI3K/AKT/mTOR signaling and promote tumorigenesis [110]. Upstream regulators of mTOR are found to be mutated in the majority of patients with non-small-cell carcinomas. Additionally, previous studies have shown that phosphorylated mTOR leads to progression of small cell lung cancer [110]. mTOR mutations have also been evident in several brain cancers. Formation of glioblastoma relies on constitutive activation of mTORC1. This increase in activity is most commonly due to loss of function of PTEN, an inhibitor of the mTOR pathway. Regardless of the original mutation leading to increased mTOR activity, mTOR plays a major role in the continued proliferation of glioblastoma [113]. Similar findings are seen in breast cancer. Breast cancer with mTOR hyperactivity is often due to PIK3CA mutation which occurs in 20–50% of breast cancers [110]. mTOR activity is often positively correlated with worse prognosis and cancer cell survival [110,113]. Given mTOR’s influence on both tumorigenesis as well as prognosis, the use of mTOR related therapies is part of standard practice. Additionally, there continues to be ongoing development of novel ways to treat such mutations.

Given the prevalence of mTOR mutations in an assortment of cancers, mTOR pathway inhibitors have been developed and are currently being used [114]. Rapalogs, a class of Rapamycin analogs used as mTOR inhibitors, share the same mechanism of action as Rapamycin by binding to FKBP12 and primarily inhibiting mTORC1, thereby reducing mTOR activation [115]. Rapalogs have been shown to have mixed success in treating a variety of cancers with mTOR mutations [116,117,118,119,120]. While there continues to be development and testing of new rapalogs, only Everolimus—used in already treated metastatic renal cell carcinoma and neuroendocrine tumors among others and temsirolimus used in treatment-resistant mantle cell lymphoma and metastatic renal cell carcinoma—are approved for use in the United States [114]. Given that rapalogs exclusively target mTORC1 there has been considerable effort to develop mTOR kinase inhibitors which can target both mTORC1 and mTORC2. These mTOR kinase inhibitors obstruct all kinase dependent functions of the two complexes [115].

Vistusertib (AZD2014), a small molecule ATP competitive inhibitor of both mTORC1 and mTORC2 was shown to be effective in vitro and in a murine model of ER + breast cancer using Everolimus-resistant cell lines [121]. This may indicate the additive effect of also inhibiting mTORC2. Several clinical trials have been conducted or are currently ongoing using Vistusertib in various cancers. One such study was a Phase 2 randomized clinical trial which combined Vistusertib with Paclitaxel vs. Paclitaxel in 140 participants with platinum-resistant ovarian carcinoma. The results showed no significant increase in progression free survival or in overall survival [122]. Despite the high prevalence of mTOR-mutated cancers, solo and combo treatments using rapalogs or mTOR kinase inhibitors have shown at best mixed results [116,117,118,119,120,122,123,124].

Given the varying success of rapalogs as well as the complex upstream and downstream effectors in the mTOR pathway dual therapy is likely to be most effective [108,109,110,114]. Clinical trials both completed and ongoing have used Rapalogs (Everolimus & Temsirolimus) in conjunction with other therapy to try and treat a wide variety of cancers. One study combined Everolimus with Vandetanib, an inhibitor of vascular endothelial growth factor receptors. This combination was used in a pediatric population, most of whom had sarcoma. The toxicity of the combo was shown to be similar to when the drugs are used individually and 3 patients had exceptional responses. After genome sequencing one of these patients was found to have ASPL/ASPSCR1-TFE3 fusion transcript. This fusion protein through a phosphorylation pathway leads to an increase in MAP kinase and the PI3K/AKT pathway. The researchers concluded that since everolimus acts downstream of PI3K/AKT it may have been an important component in this patient’s response to treatment [116]. An additional phase I trial used Temsirolimus and Erlotinib—an EGFR inhibitor to treat refractory solid tumors. EGFR can lead to phosphorylation and thus activation of PI3K which then phosphorylates the downstream mTORC1 and regulates cell growth. The study included 41 patients who took the combo therapy. While the therapy was relatively tolerable the results were mixed. Twenty-six patients had at least one scan post treatment of which 17 showed stable disease [120]. Given the varied results more research is needed into the specific mutations and biomarkers that would better predict which patients these drugs could be effective.

mTOR inhibitors (Everolimus, Temsirolimus, Ridaforolimus) are associated with a characteristic toxicity profile, most notably oral stomatitis/mucositis, rash, fatigue, and metabolic disturbances including hyperglycemia, hyperlipidemia, and hypophosphatemia, with stomatitis and metabolic derangements being the most frequent dose-limiting toxicities [125,126]. Non-infectious pneumonitis and, rarely, acute kidney injury (including acute tubular necrosis), are important but less common adverse events requiring vigilance. Stomatitis typically presents early, is usually grade 1–2, and is managed with topical corticosteroids, dose interruption, or reduction; severe cases may necessitate discontinuation. Metabolic toxicities (hyperglycemia, hyperlipidemia) are managed with regular monitoring, dietary modification, and pharmacologic intervention (e.g., statins, antihyperglycemics), with dose adjustment reserved for refractory or severe cases. Pneumonitis requires prompt recognition, exclusion of infection, and corticosteroid therapy, with discontinuation of mTOR inhibitors in severe or progressive cases. Most toxicities are reversible with supportive care and dose modification, and multidisciplinary management is recommended to optimize tolerability and maintain therapeutic efficacy.

### 4.4. PTEN

PTEN is a tumor suppressor protein with lipid phosphatase activity. Through use of this lipid phosphatase, PTEN is capable of hydrolyzing phosphatidylinositol (3,4,5)-trisphosphate (PIP3) to phosphatidylinositol (4,5)-bisphosphate (PIP2). This chemical transformation inhibits PIP3 from activating factors that promote cell growth and survival such as the PI3K/AKT pathway [127]. Since PTEN is a major regulator of cell growth, its regulation is of great importance. PTEN is found both in the nucleus and cytoplasm. In both the cytoplasm and nucleus, it leads to inactivation of AKT. In the nucleus it also associates with p53, centromere specific binding protein C, and anaphase-promoting complex. These associations result in different cellular activity but all promote cellular growth and division that lacks mutation. PTEN also decreases MAP kinase activity through dephosphorylation which results in decreased mitogenic signaling [128]. PTEN’s activity is regulated through several mechanisms such as phosphorylation, acetylation, oxidation, and ubiquitination all of which generally decrease its activity [127]. Given PTEN’s critical role in regulating cell growth it comes as no surprise that mutations of this tumor suppressor protein are a common cause of cancer formation [127,129,130].

PTEN has been shown to play a role in a variety of different types of cancer formation as well as leading to drug resistance [130]. Alterations of the PTEN gene occur in 35% of endometrial cancers, 32% of glial tumors, 17% of prostate cancers, 12% of non-small-cell lung cancer, and 9% of breast cancers [131]. A common finding of altered PTEN genes is through hypermethylation of CpG islands at the PTEN promoter site. This methylation leads to transcriptional silencing and thus the loss of inhibitory effects due to PTEN. In HER2+ breast cancer, the efficacy of Trastuzumab has been tied to whether PTEN was inhibited through NOTCH-1. In cases where there was PTEN inhibition, use of Trastuzumab can actually result in progression to a more aggressive form of cancer [129]. A study looking at germline PTEN mutations found there was an 85% lifetime risk of developing breast cancer with a PTEN mutation [130]. Glioblastoma multiforme presents similar issues in regard to PTEN. Temozolomide, which is used along with radiotherapy, has been shown to be less effective in patients who lose PTEN function. This represents a major challenge in treatment as 40% of patients lose PTEN function [129]. Similar findings have also been demonstrated in prostate cancer as PTEN is also commonly altered. Lastly, the pathogenesis of NSCLC often results in constituent AKT activation following loss of PTEN inhibition. This results in both resistance to treatment as well as the original pathogenesis of the disease [129].

To date, there are no approved therapeutic options which specifically target PTEN [132]. However, given its role in treatment resistance and important regulatory function targeted agents to the AKT-PTEN pathway are in development. Recently, Capivasertib has been given FDA approval [133]. Capivasertib is an AKT inhibitor that was recently used in a phase 3 clinical trial. In addition to Fulvestrant, Capivasertib was added to the treatment regimen in patients with HR+ advanced breast cancer with alterations in PIK3CA, AKT1, or PTEN. The dual therapy significantly increased progression free survival providing evidence for the importance of this pathway in designing future clinical treatments [134]. Capivasertib has also shown to be effective in treating germline PTEN mutations as seen in Cowden syndrome further strengthening the mechanistic relationship between PTEN and AKT [135].

Targeting of oncogenes (ex. AKT, PI3Kβ) in the PTEN pathway in PTEN-mutated cancers is currently the subject of many other clinical trials. One such example, a phase 1b/2 study used AZD8186, a selective PI3Kβ inhibitor with Paclitaxael in patients with advanced gastric cancer. In phase II of the trial 18 patients all of whom had loss of PTEN function demonstrated no significant benefits when given the combo therapy compared to just Paclitaxel. The only exception was one patient who had a PIK3CB E1051K mutation. This standout case may provide some support for the treatment but only those with specific mutations [136].

The targeting of a tumor suppressor like PTEN presents a greater challenge than targeting overactive oncogenes in the pathway such as AKT. One new method is using the concept of synthetic lethality. Synthetic lethality begins with a cell that has an inactivated tumor suppressor (ex. PTEN) and through inhibition of another gene leads to cell death. Several recent studies have looked at potential synthetic lethal interaction of PTEN as summarized in a previous review [137]. One example used was the BCR-ABL protein which is a causative factor of chronic myeloid leukemia. BCR-ABL transports PTEN to the cytoplasm causing diminished intranuclear PTEN activity. PTEN is then inactivated in the cytoplasm by Casein kinase II. This mechanism may indicate the possibility of using synthetic lethality in this case via targeting Casein kinase II to maintain PTEN activity [137].

While it may seem logical that factors increasing PTEN activity could be therapeutically valuable, preclinical studies have actually shown some evidence that PTEN inhibitors can be useful cancer treatments [138]. The proposed mechanism is that the complete loss of PTEN leads to greater activation of the PI3K/AKT pathway resulting in cell senescence through a p53 dependent process [139]. By using a mouse model with prostate tumor xenografts and the PTEN inhibitor, VO-OHpic tumorigenesis was significantly reduced [139]. In another study using VO-OHpic, PTEN inhibitors were also shown to be effective when using hepatocarcinoma cell lines [140]. Given the varied mechanisms of successful clinical and preclinical trials targeting PTEN and proteins in the pathway, further research needs to be undertaken to better elucidate how to best target patients who present PTEN-mutated cancers.

## 5. Conclusions

The ERK1/2 and PI3K signaling pathways remain significant targets in cancer therapy due to the central roles they play in driving tumorigenesis. While substantial progress has been made in developing inhibitors for these pathways, challenges such as treatment resistance call for more research and clinical trials to be undertaken. Recent research has been demonstrating that combination therapies represent a promising strategy for improving treatment outcomes. In this review, we underscored the importance of further research into the development, testing, and optimization of novel compounds and combination therapies that target the ERK1/2 and PI3K signaling pathways, as this has significant therapeutic potential for cancer patients. An overview of the targeted therapies for the ERK1/2 & PI3K pathways can be seen in Table 1 below.

## Figures and Tables

**Table 1:** Targeted Therapies for the ERK1/2 & PI3K Pathways.

Pathway Target	Agent/Combination	Class/Mechanism	Regulatory Status	Key Indications
EGFR	Osimertinib	3rd-generation EGFR TKI	FDA-approved	EGFR-mutant NSCLC
EGFR	Amivantamab	Bispecific EGFR/MET antibody	FDA-approved	NSCLC with EGFR exon 20 insertions
FGFR3	Erdafitinib	Pan-FGFR TKI	FDA-approved	FGFR-altered urothelial carcinoma
RAS	Salirasib	RAS membrane disruptor	Phase II	KRAS-mutant solid tumors
RAF + MEK	Dabrafenib + Trametinib	BRAF inhibitor + MEK inhibitor	FDA-approved combo	BRAF V600E/K melanoma
MEK	Selumetinib	MEK1/2 inhibitor	FDA-approved	NF-1 plexiform neurofibroma
ERK	Ulixertinib	ERK1/2 inhibitor	Phase II	MAPK-altered solid tumors
PI3Kα	Alpelisib	PI3Kα-selective inhibitor	FDA-approved	PIK3CA-mutant HR+/HER2- BC
Pan-PI3K	Copanlisib	Pan-class I PI3K inhibitor	FDA-approved	Relapsed follicular lymphoma
AKT	Capivasertib	Pan-AKT inhibitor	FDA-approved (2024)	HR+/HER2- BC with PIK3CA/AKT/PTEN alter.
AKT	Ipatasertib	Pan-AKT inhibitor	Phase III	TNBC; endocrine-resistant HR+ BC
mTORC1	Everolimus	Rapalog (mTORC1 inhibitor)	FDA-approved	RCC; NET; HR+/HER2- BC
mTORC1	Temsirolimus	Rapalog (mTORC1 inhibitor)	FDA-approved	Advanced RCC; mantle-cell lymphoma
mTORC1/2	Vistusertib (AZD2014)	ATP-competitive mTORC1/2 inhibitor	Phase II	Platinum-resistant ovarian CA
PI3K/mTOR	Dactolisib (BEZ235)	Dual PI3K/mTOR inhibitor	Phase II	Advanced solid tumors; leukemia
PTEN-deficient	Capivasertib + Olaparib	AKT inhibitor + PARP inhibitor	Phase I/II	PTEN-altered or HRD solid tumors

## Abbreviations

The following abbreviations are used in this manuscript:

MAPK	Mitogen activated protein kinase
PI3K	Phosphoinositide 3-kinase
EGFR	Epidermal growth factor receptor
ERK1/2	Extracellular signal-regulated kinases 1 and 2
KRAS	Kirsten rat sarcoma viral oncogene homolog
FGFR3	Fibroblast growth factor receptor 3
mTOR	Mammalian Target of Rapamycin

## References

[1] Lavoie H., Gagnon J., Therrien M. (2020). ERK signalling: A master regulator of cell behaviour, life and fate. Nat. Rev. Mol. Cell Biol..

[2] Janku F., Yap T.A., Meric-Bernstam F. (2018). Targeting the PI3K pathway in cancer: Are we making headway?. Nat. Rev. Clin. Oncol..

[3] Regad T. (2015). Targeting RTK Signaling Pathways in Cancer. Cancers.

[4] Bahar M.E., Kim H.J., Kim D.R. (2023). Targeting the RAS/RAF/MAPK pathway for cancer therapy: From mechanism to clinical studies. Signal Transduct. Target. Ther..

[5] Sirico M., D’Angelo A., Gianni C., Casadei C., Merloni F., De Giorgi U. (2023). Current State and Future Challenges for PI3K Inhibitors in Cancer Therapy. Cancers.

[6] Yang J., Nie J., Ma X., Wei Y., Peng Y., Wei X. (2019). Targeting PI3K in cancer: Mechanisms and advances in clinical trials. Mol. Cancer.

[7] Klempner S.J., Myers A.P., Cantley L.C. (2013). What a tangled web we weave: Emerging resistance mechanisms to inhibition of the phosphoinositide 3-kinase pathway. Cancer Discov..

[8] Czarnecka A.M., Bartnik E., Fiedorowicz M., Rutkowski P. (2020). Targeted Therapy in Melanoma and Mechanisms of Resistance. Int. J. Mol. Sci..

[9] McCubrey J.A., Steelman L.S., Chappell W.H., Abrams S.L., Montalto G., Cervello M., Nicoletti F., Fagone P., Malaponte G., Mazzarino M.C. (2012). Mutations and deregulation of Ras/Raf/MEK/ERK and PI3K/PTEN/Akt/mTOR cascades which alter therapy response. Oncotarget.

[10] Wee S., Jagani Z., Xiang K.X., Loo A., Dorsch M., Yao Y.M., Sellers W.R., Lengauer C., Stegmeier F. (2009). PI3K pathway activation mediates resistance to MEK inhibitors in KRAS mutant cancers. Cancer Res..

[11] Wright S.C.E., Vasilevski N., Serra V., Rodon J., Eichhorn P.J.A. (2021). Mechanisms of Resistance to PI3K Inhibitors in Cancer: Adaptive Responses, Drug Tolerance and Cellular Plasticity. Cancers.

[12] Jaiswal B.S., Durinck S., Stawiski E.W., Yin J., Wang W., Lin E., Moffat J., Martin S.E., Modrusan Z., Seshagiri S. (2018). ERK Mutations and Amplification Confer Resistance to ERK-Inhibitor Therapy. Clin. Cancer Res..

[13] Hayes T.K., Neel N.F., Hu C., Gautam P., Chenard M., Long B., Aziz M., Kassner M., Bryant K.L., Pierobon M. (2016). Long-Term ERK Inhibition in KRAS-Mutant Pancreatic Cancer Is Associated with MYC Degradation and Senescence-like Growth Suppression. Cancer Cell.

[14] Pettazzoni P., Viale A., Shah P., Carugo A., Ying H., Wang H., Genovese G., Seth S., Minelli R., Green T. (2015). Genetic events that limit the efficacy of MEK and RTK inhibitor therapies in a mouse model of KRAS-driven pancreatic cancer. Cancer Res..

[15] Lazarte J.M.S., Ofosu-Asante K., Tilghman S.L., Lamango N.S. (2025). PCAIs stimulate MAPK, PI3K/AKT pathways and ROS-Mediated apoptosis in aromatase inhibitor-resistant breast cancer cells while disrupting actin filaments and focal adhesion. Oncotarget.

[16] Hijazi M., Casado P., Akhtar N., Alvarez-Teijeiro S., Rajeeve V., Cutillas P.R. (2022). eEF2K Activity Determines Synergy to Cotreatment of Cancer Cells With PI3K and MEK Inhibitors. Mol. Cell Proteom..

[17] Atanasova V.S., Riedl A., Strobl M., Flandorfer J., Unterleuthner D., Weindorfer C., Neuhold P., Stang S., Hengstschlager M., Bergmann M. (2023). Selective Eradication of Colon Cancer Cells Harboring PI3K and/or MAPK Pathway Mutations in 3D Culture by Combined PI3K/AKT/mTOR Pathway and MEK Inhibition. Int. J. Mol. Sci..

[18] Sigismund S., Avanzato D., Lanzetti L. (2018). Emerging functions of the EGFR in cancer. Mol. Oncol..

[19] Kovacs E., Zorn J.A., Huang Y., Barros T., Kuriyan J. (2015). A structural perspective on the regulation of the epidermal growth factor receptor. Annu. Rev. Biochem..

[20] Yuan M., Huang L.L., Chen J.H., Wu J., Xu Q. (2019). The emerging treatment landscape of targeted therapy in non-small-cell lung cancer. Signal Transduct. Target. Ther..

[21] Voldborg B.R., Damstrup L., Spang-Thomsen M., Poulsen H.S. (1997). Epidermal growth factor receptor (EGFR) and EGFR mutations, function and possible role in clinical trials. Ann. Oncol..

[22] Hsu P.C., Jablons D.M., Yang C.T., You L. (2019). Epidermal Growth Factor Receptor (EGFR) Pathway, Yes-Associated Protein (YAP) and the Regulation of Programmed Death-Ligand 1 (PD-L1) in Non-Small Cell Lung Cancer (NSCLC). Int. J. Mol. Sci..

[23] Seto E.S., Bellen H.J., Lloyd T.E. (2002). When cell biology meets development: Endocytic regulation of signaling pathways. Genes. Dev..

[24] Harrison P.T., Vyse S., Huang P.H. (2020). Rare epidermal growth factor receptor (EGFR) mutations in non-small cell lung cancer. Semin. Cancer Biol..

[25] Janani B., Vijayakumar M., Priya K., Kim J.H., Prabakaran D.S., Shahid M., Al-Ghamdi S., Alsaidan M., Othman Bahakim N., Hassan Abdelzaher M. (2022). EGFR-Based Targeted Therapy for Colorectal Cancer-Promises and Challenges. Vaccines.

[26] Jung S., Kim D.H., Choi Y.J., Kim S.Y., Park H., Lee H., Choi C.M., Sung Y.H., Lee J.C., Rho J.K. (2021). Contribution of p53 in sensitivity to EGFR tyrosine kinase inhibitors in non-small cell lung cancer. Sci. Rep..

[27] Liu Q., Yu S., Zhao W., Qin S., Chu Q., Wu K. (2018). EGFR-TKIs resistance via EGFR-independent signaling pathways. Mol. Cancer.

[28] Patel S., Patel J.D. (2023). Current and Emerging Treatment Options for Patients with Metastatic EGFR-Mutated Non-small Cell Lung Cancer After Progression on Osimertinib and Platinum-Based Chemotherapy: A Podcast Discussion. Adv. Ther..

[29] Divan H.A., Bittoni M.A., Krishna A., Carbone D.P. (2023). Real-world treatment patterns and outcomes of patients with metastatic nonsquamous non-small cell lung cancer after progression on standard-of-care therapy in the United States. Lung Cancer.

[30] Johnson M., Garassino M.C., Mok T., Mitsudomi T. (2022). Treatment strategies and outcomes for patients with EGFR-mutant non-small cell lung cancer resistant to EGFR tyrosine kinase inhibitors: Focus on novel therapies. Lung Cancer.

[31] Yu H.A., Goto Y., Hayashi H., Felip E., Chih-Hsin Yang J., Reck M., Yoh K., Lee S.H., Paz-Ares L., Besse B. (2023). HERTHENA-Lung01, a Phase II Trial of Patritumab Deruxtecan (HER3-DXd) in Epidermal Growth Factor Receptor-Mutated Non-Small-Cell Lung Cancer After Epidermal Growth Factor Receptor Tyrosine Kinase Inhibitor Therapy and Platinum-Based Chemotherapy. J. Clin. Oncol..

[32] Araki T., Kanda S., Horinouchi H., Ohe Y. (2023). Current treatment strategies for EGFR-mutated non-small cell lung cancer: From first line to beyond osimertinib resistance. Jpn. J. Clin. Oncol..

[33] Lim S.M., Fujino T., Kim C., Lee G., Lee Y.H., Kim D.W., Ahn J.S., Mitsudomi T., Jin T., Lee S.Y. (2023). BBT-176, a Novel Fourth-Generation Tyrosine Kinase Inhibitor for Osimertinib-Resistant EGFR Mutations in Non-Small Cell Lung Cancer. Clin. Cancer Res..

[34] Cho B.C., Lu S., Felip E., Spira A.I., Girard N., Lee J.S., Lee S.H., Ostapenko Y., Danchaivijitr P., Liu B. (2024). Amivantamab plus Lazertinib in Previously Untreated EGFR-Mutated Advanced NSCLC. N. Engl. J. Med..

[35] Zhou C., Tang K.J., Cho B.C., Liu B., Paz-Ares L., Cheng S., Kitazono S., Thiagarajan M., Goldman J.W., Sabari J.K. (2023). Amivantamab plus Chemotherapy in NSCLC with EGFR Exon 20 Insertions. N. Engl. J. Med..

[36] Yan H., Tang S., Tang S., Zhang J., Guo H., Qin C., Hu H., Zhong C., Yang L., Zhu Y. (2022). miRNAs in anti-cancer drug resistance of non-small cell lung cancer: Recent advances and future potential. Front. Pharmacol..

[37] Manojmouli C., Pasha T.Y., Rahamathulla M., H P G., B L K., K M G., K N P., Hussain S.M., Ahmed M.M., Shivanandappa T.B. (2025). Epidermal growth factor receptors unveiled: A comprehensive survey on mutations, clinical insights of global inhibitors, and emergence of heterocyclic derivatives as EGFR inhibitors. J. Drug Target..

[38] Dickerson H., Diab A., Al Musaimi O. (2024). Epidermal Growth Factor Receptor Tyrosine Kinase Inhibitors in Cancer: Current Use and Future Prospects. Int. J. Mol. Sci..

[39] Shaban N., Kamashev D., Emelianova A., Buzdin A. (2023). Targeted Inhibitors of EGFR: Structure, Biology, Biomarkers, and Clinical Applications. Cells.

[40] Halder S., Basu S., Lall S.P., Ganti A.K., Batra S.K., Seshacharyulu P. (2023). Targeting the EGFR signaling pathway in cancer therapy: What’s new in 2023?. Expert Opin. Ther. Targets.

[41] Zheng J., Zhang W., Li L., He Y., Wei Y., Dang Y., Nie S., Guo Z. (2022). Signaling Pathway and Small-Molecule Drug Discovery of FGFR: A Comprehensive Review. Front. Chem..

[42] Alberca-Del Arco F., Prieto-Cuadra D., Santos-Perez de la Blanca R., Saez-Barranquero F., Matas-Rico E., Herrera-Imbroda B. (2024). New Perspectives on the Role of Liquid Biopsy in Bladder Cancer: Applicability to Precision Medicine. Cancers.

[43] Andreozzi F., Dragani M., Quivoron C., Le Bras F., Assi T., Danu A., Belhadj K., Lazarovici J., Cotteret S., Bernard O.A. (2023). Precision Medicine Approach Based on Molecular Alterations for Patients with Relapsed or Refractory Multiple Myeloma: Results from the MM-EP1 Study. Cancers.

[44] Du S., Zhang Y., Xu J. (2023). Current progress in cancer treatment by targeting FGFR signaling. Cancer Biol. Med..

[45] Benjamin D.J., Hsu R. (2023). Treatment approaches for FGFR-altered urothelial carcinoma: Targeted therapies and immunotherapy. Front. Immunol..

[46] Xiao J.F., Caliri A.W., Duex J.E., Theodorescu D. (2021). Targetable Pathways in Advanced Bladder Cancer: FGFR Signaling. Cancers.

[47] Sternberg C.N., Petrylak D.P., Bellmunt J., Nishiyama H., Necchi A., Gurney H., Lee J.L., van der Heijden M.S., Rosenbaum E., Penel N. (2023). FORT-1: Phase II/III Study of Rogaratinib Versus Chemotherapy in Patients with Locally Advanced or Metastatic Urothelial Carcinoma Selected Based on FGFR1/3 mRNA Expression. J. Clin. Oncol..

[48] Katoh M., Loriot Y., Brandi G., Tavolari S., Wainberg Z.A., Katoh M. (2024). FGFR-targeted therapeutics: Clinical activity, mechanisms of resistance and new directions. Nat. Rev. Clin. Oncol..

[49] Conroy M., Cowzer D., Kolch W., Duffy A.G. (2021). Emerging RAS-directed therapies for cancer. Cancer Drug Resist..

[50] Vasan N., Boyer J.L., Herbst R.S. (2014). A RAS renaissance: Emerging targeted therapies for KRAS-mutated non-small cell lung cancer. Clin. Cancer Res..

[51] Chen K., Zhang Y., Qian L., Wang P. (2021). Emerging strategies to target RAS signaling in human cancer therapy. J. Hematol. Oncol..

[52] Liu C., Ye D., Yang H., Chen X., Su Z., Li X., Ding M., Liu Y. (2023). RAS-targeted cancer therapy: Advances in drugging specific mutations. MedComm.

[53] Tatli O., Dinler Doganay G. (2021). Recent Developments in Targeting RAS Downstream Effectors for RAS-Driven Cancer Therapy. Molecules.

[54] Poulikakos P.I., Sullivan R.J., Yaeger R. (2022). Molecular Pathways and Mechanisms of BRAF in Cancer Therapy. Clin. Cancer Res..

[55] Lebrun H., Turpin A., Zerbib P. (2021). Therapeutic implications of B-RAF mutations in colorectal cancer. J. Visc. Surg..

[56] Alese O.B., Wu C., Chapin W.J., Ulanja M.B., Zheng-Lin B., Amankwah M., Eads J. (2023). Update on Emerging Therapies for Advanced Colorectal Cancer. Am. Soc. Clin. Oncol. Educ. Book.

[57] Owsley J., Stein M.K., Porter J., In G.K., Salem M., O’Day S., Elliott A., Poorman K., Gibney G., VanderWalde A. (2021). Prevalence of class I-III BRAF mutations among 114,662 cancer patients in a large genomic database. Exp Biol Med.

[58] Laha D., Nilubol N., Boufraqech M. (2020). New Therapies for Advanced Thyroid Cancer. Front. Endocrinol..

[59] Okubagzhi G.S. (1988). Fulfilling the potential of traditional birth attendants. World Health Forum.

[60] Neuzillet C., Tijeras-Raballand A., de Mestier L., Cros J., Faivre S., Raymond E. (2014). MEK in cancer and cancer therapy. Pharmacol. Ther..

[61] Wu Z.P., Wang Y.L., Wang L.C., Liu Z.Y., Fan R.R., Zan X., Liang R.C., Yang J.L., Zhou L.X., Xu J.G. (2023). Case Report: Successful Use of BRAF/MEK Inhibitors in Aggressive BRAF-mutant Craniopharyngioma. World Neurosurg..

[62] Gu J., Yao W., Shi P., Zhang G., Owonikoko T.K., Ramalingam S.S., Sun S.Y. (2020). MEK or ERK inhibition effectively abrogates emergence of acquired osimertinib resistance in the treatment of epidermal growth factor receptor-mutant lung cancers. Cancer.

[63] Subbiah V., Baik C., Kirkwood J.M. (2020). Clinical Development of BRAF plus MEK Inhibitor Combinations. Trends Cancer.

[64] Welsh S.J., Rizos H., Scolyer R.A., Long G.V. (2016). Resistance to combination BRAF and MEK inhibition in metastatic melanoma: Where to next?. Eur. J. Cancer.

[65] Goodman R.S., Di Guardo L., Maurichi A., Kirwin B., Khattak A., Vanella V., Lee J., Lawless A., Czapla J., Spagnoletti A. (2023). Long-term outcomes and persistent toxicities following BRAF/MEK inhibitor therapy for advanced melanoma. Eur. J. Cancer.

[66] Datta J., Dai X., Bianchi A., De Castro Silva I., Mehra S., Garrido V.T., Lamichhane P., Singh S.P., Zhou Z., Dosch A.R. (2022). Combined MEK and STAT3 Inhibition Uncovers Stromal Plasticity by Enriching for Cancer-Associated Fibroblasts with Mesenchymal Stem Cell-Like Features to Overcome Immunotherapy Resistance in Pancreatic Cancer. Gastroenterology.

[67] Senechal I., Andres M.S., Tong J., Ramalingam S., Nazir M.S., Rosen S.D., Young K., Idaikkadar P., Larkin J., Lyon A.R. (2024). Risk Stratification, Screening and Treatment of BRAF/MEK Inhibitors-Associated Cardiotoxicity. Curr. Oncol. Rep..

[68] Mendez-Martinez S., Calvo P., Ruiz-Moreno O., Pardinas Baron N., Lecinena Bueno J., Gil Ruiz M.D.R., Pablo L. (2019). Ocular Adverse Events Associated with Mek Inhibitors. Retina.

[69] Friedland R., Glick M., Amitay-Laish I., Toledano H. (2024). Cutaneous Reactions in Pediatric Patients Treated with MEK Inhibitors: A Retrospective Single-Center Study. Dermatology.

[70] Abdel-Rahman O., ElHalawani H., Ahmed H. (2015). Risk of selected dermatological toxicities in cancer patients treated with MEK inhibitors: A comparative systematic review and meta-analysis. Future Oncol..

[71] Jeng-Miller K.W., Miller M.A., Heier J.S. (2024). Ocular Effects of MEK Inhibitor Therapy: Literature Review, Clinical Presentation, and Best Practices for Mitigation. Oncologist.

[72] Heinzerling L., Eigentler T.K., Fluck M., Hassel J.C., Heller-Schenck D., Leipe J., Pauschinger M., Vogel A., Zimmer L., Gutzmer R. (2019). Tolerability of BRAF/MEK inhibitor combinations: Adverse event evaluation and management. ESMO Open.

[73] Mourad N., Lourenco N., Delyon J., Eftekhari P., Bertheau P., Allayous C., Ballon A., Pages C., Allez M., Lebbe C. (2019). Severe gastrointestinal toxicity of MEK inhibitors. Melanoma Res..

[74] Iriarte C., Yeh J.E., Alloo A., Boull C., Carlberg V.M., Coughlin C.C., Lara-Corrales I., Levy R., Nguyen C.V., Oza V.S. (2024). Mucocutaneous toxicities from MEK inhibitors: A scoping review of the literature. Support. Care Cancer.

[75] Garutti M., Bergnach M., Polesel J., Palmero L., Pizzichetta M.A., Puglisi F. (2022). BRAF and MEK Inhibitors and Their Toxicities: A Meta-Analysis. Cancers.

[76] Li Q., Li Z., Luo T., Shi H. (2022). Targeting the PI3K/AKT/mTOR and RAF/MEK/ERK pathways for cancer therapy. Mol. Biomed..

[77] Serra V., Scaltriti M., Prudkin L., Eichhorn P.J., Ibrahim Y.H., Chandarlapaty S., Markman B., Rodriguez O., Guzman M., Rodriguez S. (2011). PI3K inhibition results in enhanced HER signaling and acquired ERK dependency in HER2-overexpressing breast cancer. Oncogene.

[78] Chen P., Xu W., Luo Y., Zhang Y., He Y., Yang S., Yuan Z. (2017). MicroRNA 543 suppresses breast cancer cell proliferation, blocks cell cycle and induces cell apoptosis via direct targeting of ERK/MAPK. Onco. Targets Ther..

[79] Ramirez A., Boulaiz H., Morata-Tarifa C., Peran M., Jimenez G., Picon-Ruiz M., Agil A., Cruz-Lopez O., Conejo-Garcia A., Campos J.M. (2014). HER2-signaling pathway, JNK and ERKs kinases, and cancer stem-like cells are targets of Bozepinib small compound. Oncotarget.

[80] Krysan K., Reckamp K.L., Dalwadi H., Sharma S., Rozengurt E., Dohadwala M., Dubinett S.M. (2005). Prostaglandin E2 activates mitogen-activated protein kinase/ERK pathway signaling and cell proliferation in non-small cell lung cancer cells in an epidermal growth factor receptor-independent manner. Cancer Res..

[81] Huang Y., Zhen Y., Chen Y., Sui S., Zhang L. (2023). Unraveling the interplay between RAS/RAF/MEK/ERK signaling pathway and autophagy in cancer: From molecular mechanisms to targeted therapy. Biochem. Pharmacol..

[82] Zhang M., Bai Y., Xu C., Qi Y., Meng J., Zhang W., Su H., Yan W. (2021). Blockage of Extracellular Signal-Regulated Kinase Exerts an Antitumor Effect via Regulating Energy Metabolism and Enhances the Efficacy of Autophagy Inhibitors by Regulating Transcription Factor EB Nuclear Translocation in Osteosarcoma. Front. Cell Dev. Biol..

[83] Pan X., Pei J., Wang A., Shuai W., Feng L., Bu F., Zhu Y., Zhang L., Wang G., Ouyang L. (2022). Development of small molecule extracellular signal-regulated kinases (ERKs) inhibitors for cancer therapy. Acta Pharm. Sin. B.

[84] Smalley I., Smalley K.S.M. (2018). ERK Inhibition: A New Front in the War against MAPK Pathway-Driven Cancers?. Cancer Discov..

[85] Fu L., Chen S., He G., Chen Y., Liu B. (2022). Targeting Extracellular Signal-Regulated Protein Kinase 1/2 (ERK1/2) in Cancer: An Update on Pharmacological Small-Molecule Inhibitors. J. Med. Chem..

[86] Wu J., Liu D., Offin M., Lezcano C., Torrisi J.M., Brownstein S., Hyman D.M., Gounder M.M., Abida W., Drilon A. (2021). Characterization and management of ERK inhibitor associated dermatologic adverse events: Analysis from a nonrandomized trial of ulixertinib for advanced cancers. Investig. New Drugs.

[87] Roskoski R. (2019). Corrigendum to Targeting ERK1/2 protein-serine/threonine kinases in human cancers [Pharmcol. Res. 142 (2019) 151–168]. Pharmacol. Res..

[88] Belair D.G., Sudak K., Connelly K., Collins N.D., Kopytek S.J., Kolaja K.L. (2021). Investigation Into the Role of ERK in Tyrosine Kinase Inhibitor-Induced Neuropathy. Toxicol. Sci..

[89] Castel P., Toska E., Engelman J.A., Scaltriti M. (2021). The present and future of PI3K inhibitors for cancer therapy. Nat. Cancer.

[90] Zhang Z., Richmond A. (2021). The Role of PI3K Inhibition in the Treatment of Breast Cancer, Alone or Combined with Immune Checkpoint Inhibitors. Front. Mol. Biosci..

[91] Glaviano A., Foo A.S.C., Lam H.Y., Yap K.C.H., Jacot W., Jones R.H., Eng H., Nair M.G., Makvandi P., Geoerger B. (2023). PI3K/AKT/mTOR signaling transduction pathway and targeted therapies in cancer. Mol. Cancer.

[92] Yu M., Chen J., Xu Z., Yang B., He Q., Luo P., Yan H., Yang X. (2023). Development and safety of PI3K inhibitors in cancer. Arch. Toxicol..

[93] Markham A. (2019). Alpelisib: First Global Approval. Drugs.

[94] Sabbah D.A., Hajjo R., Bardaweel S.K., Zhong H.A. (2024). Targeting the PI3K/AKT signaling pathway in anticancer research: A recent update on inhibitor design and clinical trials (2020–2023). Expert Opin. Ther. Pat..

[95] Mayer I.A., Arteaga C.L. (2016). The PI3K/AKT Pathway as a Target for Cancer Treatment. Annu. Rev. Med..

[96] Nunnery S.E., Mayer I.A. (2019). Management of toxicity to isoform alpha-specific PI3K inhibitors. Ann. Oncol..

[97] Esposito A., Viale G., Curigliano G. (2019). Safety, Tolerability, and Management of Toxic Effects of Phosphatidylinositol 3-Kinase Inhibitor Treatment in Patients with Cancer: A Review. JAMA Oncol..

[98] Hanlon A., Brander D.M. (2020). Managing toxicities of phosphatidylinositol-3-kinase (PI3K) inhibitors. Hematol. Am. Soc. Hematol. Educ. Program..

[99] Lin X., Zhang Y., Huang H., Zhuang W., Wu L. (2025). Post-marketing safety concern of PI3K inhibitors in the cancer therapies: An 8-year disproportionality analysis from the FDA Adverse Event Reporting System. Expert Opin. Drug Saf..

[100] Drullinsky P.R., Hurvitz S.A. (2020). Mechanistic basis for PI3K inhibitor antitumor activity and adverse reactions in advanced breast cancer. Breast Cancer Res. Treat..

[101] Turner N.C., Im S.A., Saura C., Juric D., Loibl S., Kalinsky K., Schmid P., Loi S., Sunpaweravong P., Musolino A. (2024). Inavolisib-Based Therapy in PIK3CA-Mutated Advanced Breast Cancer. N. Engl. J. Med..

[102] Shariati M., Meric-Bernstam F. (2019). Targeting AKT for cancer therapy. Expert Opin. Investig. Drugs.

[103] Cheung M., Testa J.R. (2013). Diverse mechanisms of AKT pathway activation in human malignancy. Curr. Cancer Drug Targets.

[104] Manning B.D., Toker A. (2017). AKT/PKB Signaling: Navigating the Network. Cell.

[105] Hua H., Zhang H., Chen J., Wang J., Liu J., Jiang Y. (2021). Targeting AKT in cancer for precision therapy. J. Hematol. Oncol..

[106] Song M., Bode A.M., Dong Z., Lee M.H. (2019). AKT as a Therapeutic Target for Cancer. Cancer Res..

[107] Martorana F., Motta G., Pavone G., Motta L., Stella S., Vitale S.R., Manzella L., Vigneri P. (2021). AKT Inhibitors: New Weapons in the Fight Against Breast Cancer?. Front. Pharmacol..

[108] Saxton R.A., Sabatini D.M. (2017). mTOR Signaling in Growth, Metabolism, and Disease. Cell.

[109] Panwar V., Singh A., Bhatt M., Tonk R.K., Azizov S., Raza A.S., Sengupta S., Kumar D., Garg M. (2023). Multifaceted role of mTOR (mammalian target of rapamycin) signaling pathway in human health and disease. Signal Transduct. Target. Ther..

[110] Tian T., Li X., Zhang J. (2019). mTOR Signaling in Cancer and mTOR Inhibitors in Solid Tumor Targeting Therapy. Int. J. Mol. Sci..

[111] Peng Y., Wang Y., Zhou C., Mei W., Zeng C. (2022). PI3K/Akt/mTOR Pathway and Its Role in Cancer Therapeutics: Are We Making Headway?. Front. Oncol..

[112] Riquelme I., Tapia O., Espinoza J.A., Leal P., Buchegger K., Sandoval A., Bizama C., Araya J.C., Peek R.M., Roa J.C. (2016). The Gene Expression Status of the PI3K/AKT/mTOR Pathway in Gastric Cancer Tissues and Cell Lines. Pathol. Oncol. Res..

[113] Singh S., Barik D., Lawrie K., Mohapatra I., Prasad S., Naqvi A.R., Singh A., Singh G. (2023). Unveiling Novel Avenues in mTOR-Targeted Therapeutics: Advancements in Glioblastoma Treatment. Int. J. Mol. Sci..

[114] Conciatori F., Ciuffreda L., Bazzichetto C., Falcone I., Pilotto S., Bria E., Cognetti F., Milella M. (2018). mTOR Cross-Talk in Cancer and Potential for Combination Therapy. Cancers.

[115] Ballou L.M., Lin R.Z. (2008). Rapamycin and mTOR kinase inhibitors. J. Chem. Biol..

[116] Phadnis S., Wang X., Daw N.C., Herzog C.E., Subbiah I.M., Zaky W., Gouda M.A., Morani A.C., Amini B., Harrison D.J. (2023). Everolimus in combination with vandetanib in children, adolescents, and young adults: A phase I study. ESMO Open.

[117] Gordon E.M., Angel N.L., Omelchenko N., Chua-Alcala V.S., Moradkhani A., Quon D., Wong S. (2023). A Phase I/II Investigation of Safety and Efficacy of Nivolumab and nab-Sirolimus in Patients with a Variety of Tumors with Genetic Mutations in the mTOR Pathway. Anticancer Res..

[118] Nathan C.O., Hayes D.N., Karrison T., Harismendy O., Flores J.M., Moore-Medlin T., Vokes E.E., Gutkind J.S., Neupane P., Mills G. (2022). A Randomized Multi-institutional Phase II Trial of Everolimus as Adjuvant Therapy in Patients with Locally Advanced Squamous Cell Cancer of the Head and Neck. Clin. Cancer Res..

[119] Adib E., Klonowska K., Giannikou K., Do K.T., Pruitt-Thompson S., Bhushan K., Milstein M.I., Hedglin J., Kargus K.E., Sholl L.M. (2021). Phase II Clinical Trial of Everolimus in a Pan-Cancer Cohort of Patients with mTOR Pathway Alterations. Clin. Cancer Res..

[120] Park H., Williams K., Trikalinos N.A., Larson S., Tan B., Waqar S., Suresh R., Morgensztern D., Van Tine B.A., Govindan R. (2021). A phase I trial of temsirolimus and erlotinib in patients with refractory solid tumors. Cancer Chemother. Pharmacol..

[121] Guichard S.M., Curwen J., Bihani T., D’Cruz C.M., Yates J.W., Grondine M., Howard Z., Davies B.R., Bigley G., Klinowska T. (2015). AZD2014, an Inhibitor of mTORC1 and mTORC2, Is Highly Effective in ER+ Breast Cancer When Administered Using Intermittent or Continuous Schedules. Mol. Cancer Ther..

[122] Banerjee S., Giannone G., Clamp A.R., Ennis D.P., Glasspool R.M., Herbertson R., Krell J., Riisnaes R., Mirza H.B., Cheng Z. (2023). Efficacy and Safety of Weekly Paclitaxel Plus Vistusertib vs. Paclitaxel Alone in Patients with Platinum-Resistant Ovarian High-Grade Serous Carcinoma: The OCTOPUS Multicenter, Phase 2, Randomized Clinical Trial. JAMA Oncol..

[123] Hong D.S., Moore K.N., Bendell J.C., Karp D.D., Wang J.S., Ulahannan S.V., Jones S., Wu W., Donoho G.P., Ding Y. (2021). Preclinical Evaluation and Phase Ib Study of Prexasertib, a CHK1 Inhibitor, and Samotolisib (LY3023414), a Dual PI3K/mTOR Inhibitor. Clin. Cancer Res..

[124] Sen S., Tanaka R., Khatua S., Zaky W., Janku F., Penas-Prado M., Weathers S.P., Behrang A., Roszik J., Subbiah V. (2020). Dual inhibition of BRAF and mTOR in BRAF (V600E)-mutant pediatric, adolescent, and young adult brain tumors. Cold Spring Harb. Mol. Case Stud..

[125] Martins F., de Oliveira M.A., Wang Q., Sonis S., Gallottini M., George S., Treister N. (2013). A review of oral toxicity associated with mTOR inhibitor therapy in cancer patients. Oral Oncol..

[126] Borders E.B., Bivona C., Medina P.J. (2010). Mammalian target of rapamycin: Biological function and target for novel anticancer agents. Am. J. Health Syst. Pharm..

[127] Hopkins B.D., Hodakoski C., Barrows D., Mense S.M., Parsons R.E. (2014). PTEN function: The long and the short of it. Trends Biochem. Sci..

[128] Chen C.Y., Chen J., He L., Stiles B.L. (2018). PTEN: Tumor Suppressor and Metabolic Regulator. Front. Endocrinol..

[129] Luongo F., Colonna F., Calapa F., Vitale S., Fiori M.E., De Maria R. (2019). PTEN Tumor-Suppressor: The Dam of Stemness in Cancer. Cancers.

[130] Tan M.H., Mester J.L., Ngeow J., Rybicki L.A., Orloff M.S., Eng C. (2012). Lifetime cancer risks in individuals with germline PTEN mutations. Clin. Cancer Res..

[131] Fusco N., Sajjadi E., Venetis K., Gaudioso G., Lopez G., Corti C., Rocco E.G., Criscitiello C., Malapelle U., Invernizzi M. (2020). PTEN Alterations and Their Role in Cancer Management: Are We Making Headway on Precision Medicine?. Genes.

[132] Alfieri R., Giovannetti E., Bonelli M., Cavazzoni A. (2017). New Treatment Opportunities in Phosphatase and Tensin Homolog (PTEN)-Deficient Tumors: Focus on PTEN/Focal Adhesion Kinase Pathway. Front. Oncol..

[133] Nierengarten M.B. (2024). FDA approves capivasertib with fulvestrant for breast cancer. Cancer.

[134] Turner N.C., Oliveira M., Howell S.J., Dalenc F., Cortes J., Gomez Moreno H.L., Hu X., Jhaveri K., Krivorotko P., Loibl S. (2023). Capivasertib in Hormone Receptor-Positive Advanced Breast Cancer. N. Engl. J. Med..

[135] Kingston B., Bailleux C., Delaloge S., Schiavon G., Scott V., Lacroix-Triki M., Carr T.H., Kozarewa I., Gevensleben H., Kemp Z. (2019). Exceptional Response to AKT Inhibition in Patients with Breast Cancer and Germline PTEN Mutations. JCO Precis. Oncol..

[136] Suh K.J., Ryu M.H., Zang D.Y., Bae W.K., Lee H.S., Oh H.J., Kang M., Kim J.W., Kim B.J., Mortimer P.G.S. (2023). AZD8186 in Combination with Paclitaxel in Patients with Advanced Gastric Cancer: Results from a Phase Ib/II Study (KCSG ST18-20). Oncologist.

[137] Ertay A., Ewing R.M., Wang Y. (2023). Synthetic lethal approaches to target cancers with loss of PTEN function. Genes. Dis..

[138] Pulido R. (2018). PTEN Inhibition in Human Disease Therapy. Molecules.

[139] Alimonti A., Nardella C., Chen Z., Clohessy J.G., Carracedo A., Trotman L.C., Cheng K., Varmeh S., Kozma S.C., Thomas G. (2010). A novel type of cellular senescence that can be enhanced in mouse models and human tumor xenografts to suppress prostate tumorigenesis. J. Clin. Investig..

[140] Augello G., Puleio R., Emma M.R., Cusimano A., Loria G.R., McCubrey J.A., Montalto G., Cervello M. (2016). A PTEN inhibitor displays preclinical activity against hepatocarcinoma cells. Cell Cycle.

